# Model-based evaluation of admission screening strategies for the detection and control of carbapenemase-producing Enterobacterales in the English hospital setting

**DOI:** 10.1186/s12916-023-03007-1

**Published:** 2023-12-12

**Authors:** Diane Pople, Theodore Kypraios, Tjibbe Donker, Nicole Stoesser, Anna C. Seale, Ryan George, Andrew Dodgson, Rachel Freeman, Russell Hope, Ann Sarah Walker, Susan Hopkins, Julie Robotham

**Affiliations:** 1 HCAI, Fungal, AMR, AMU & Sepsis Division, UK Health Security Agency, 61 Colindale Avenue, London, NW9 5EQ UK; 2 School of Mathematical Sciences, University Park, University of Nottingham, Nottingham, NG7 2RD UK; 3 University Medical Center Freiburg, Institute for Infection Prevention and Hospital Epidemiology, Breisacher Strasse, 79106 Freiburg im Breisgau, Germany; 4 Nuffield Department of Medicine, University of Oxford, Oxford, UK; 5 NIHR Health Protection Research Unit in Antimicrobial Resistance and Healthcare Associated Infections, University of Oxford and UKHSA, Oxford, UK; 6 University of Warwick, Warwick, UK; 7 London School of Hygiene & Tropical Medicine, London, UK; 8 UK Health Security Agency, London, UK; 9 Manchester University NHS Foundation Trust, Manchester, UK; 10 UK Health Security Agency, Manchester Public Health Laboratory, Manchester Royal Infirmary, Oxford Road, Manchester, M13 9WL UK; 11 IQVIA, The Point, 37 North Wharf Road, London, W2 1AF UK; 12 UK Health Security Agency, 61 Colindale Avenue, London, NW9 5EQ UK; 13 Division of Infection and Immunity, UCL, Gower St, London, UK

**Keywords:** Carbapenemase-producing Enterobacterales, Screening, Mathematical model, Nosocomial transmission

## Abstract

**Background:**

Globally, detections of carbapenemase-producing Enterobacterales (CPE) colonisations and infections are increasing. The spread of these highly resistant bacteria poses a serious threat to public health. However, understanding of CPE transmission and evidence on effectiveness of control measures is severely lacking. This paper provides evidence to inform effective admission screening protocols, which could be important in controlling nosocomial CPE transmission.

**Methods:**

CPE transmission within an English hospital setting was simulated with a data-driven individual-based mathematical model. This model was used to evaluate the ability of the 2016 England CPE screening recommendations, and of potential alternative protocols, to identify patients with CPE-colonisation on admission (including those colonised during previous stays or from elsewhere). The model included nosocomial transmission from colonised and infected patients, as well as environmental contamination. Model parameters were estimated using primary data where possible, including estimation of transmission using detailed epidemiological data within a Bayesian framework. Separate models were parameterised to represent hospitals in English areas with low and high CPE risk (based on prevalence).

**Results:**

The proportion of truly colonised admissions which met the 2016 screening criteria was 43% in low-prevalence and 54% in high-prevalence areas respectively. Selection of CPE carriers for screening was improved in low-prevalence areas by adding readmission as a screening criterion, which doubled how many colonised admissions were selected. A minority of CPE carriers were confirmed as CPE positive during their hospital stay (10 and 14% in low- and high-prevalence areas); switching to a faster screening test pathway with a single-swab test (rather than three swab regimen) increased the overall positive predictive value with negligible reduction in negative predictive value.

**Conclusions:**

Using a novel within-hospital CPE transmission model, this study assesses CPE admission screening protocols, across the range of CPE prevalence observed in England. It identifies protocol changes—adding readmissions to screening criteria and a single-swab test pathway—which could detect similar numbers of CPE carriers (or twice as many in low CPE prevalence areas), but faster, and hence with lower demand on pre-emptive infection-control resources. Study findings can inform interventions to control this emerging threat, although further work is required to understand within-hospital transmission sources.

**Supplementary Information:**

The online version contains supplementary material available at 10.1186/s12916-023-03007-1.

## Background

Carbapenems are a class of antibiotic used to treat life-threatening infections including those caused by multidrug-resistant Gram-negative bacteria. Recent years have seen the international spread of Enterobacterales (an order of Gram-negative bacteria) which have acquired the ability to produce carbapenemase enzymes, conferring resistance to carbapenems [1, 2].

In England, detections of carbapenemase-producing Enterobacterales (CPE) colonisations and infections from patients increased every year since 2006 until 2018 (most recent year available under same reporting criteria) [3]. All regions in England have reported CPE, but resistance mechanisms (such as KPC, NDM and OXA-48-like) and prevalence are geographically heterogeneous [4] with some regions and facilities experiencing endemic CPE transmission and/or outbreaks [5–9] whereas in others the problem is less established. In response to this threat to public health, recommendations were published in 2016 on the detection and control of CPE within acute settings (hereafter referred to as the ‘Toolkit’ [10]). In October 2020, ‘acquired carbapenemase-producing Gram-negative bacteria’ were added to the list of notifiable organisms (NOIDS) and revised recommendations published for both acute and non-acute settings (‘Framework of actions to contain carbapenemase-producing Enterobacterales’, hereafter referred to as the ‘Framework’ [11]).

However, evidence on effectiveness of control measures and screening strategies is lacking and unknowns remain regarding transmission dynamics. Some UK studies have investigated the impact of test choice and timing within the screening process [12–14], reporting either improved or neutral effects on reducing patient time in the admission screening process via the adoption of PCR tests and/or removing repeat testing. The available evidence has been drawn from studies either in settings at the higher end of the range of prevalence observed in England, or from global locations where CPE is well-established [15–17]. The 2020 Framework [11] recommended that local epidemiology be considered in the development of screening criteria, but evidence addressing effectiveness and efficiency of screening strategies within the heterogeneous context observed in England, especially low-prevalence regions, is lacking.

### Role for this model

Mathematical models have been used to investigate multiple aspects of CPE dynamics and management, such as estimating the burden of colonisation [18–20] and the clinical impact or cost-effectiveness of screening [12, 21, 22], surveillance [23, 24] and infection prevention and control (IPC) strategies [25–28]. These models have proved to be a useful tool to evaluate the effects of interventions, because they can simulate the underlying, typically unobserved, processes in infectious disease dynamics. However, understanding how interventions may act given local circumstances may be difficult to infer from models parameterised from other localities, due to the heterogeneity of CPE across geographical locations—both globally and nationally. Additionally, the recommendations in England include pre-emptive enhanced IPC for patients during the admission screening process, which may limit the generalisability of existing models in which the screening informs the subsequent application of in-hospital interventions such as contact precautions [27] or decolonisation [28].

Here we evaluate efficiency and effectiveness of different admission screening protocols for hospitals in England, including the effects of patient movements and within-hospital transmission on the protocol performance. We consider both the admission screening selection criteria and the screening test pathway to help inform decisions on the best strategy to implement in different settings.

## Methods

### Model structure

**Fig. 1:**
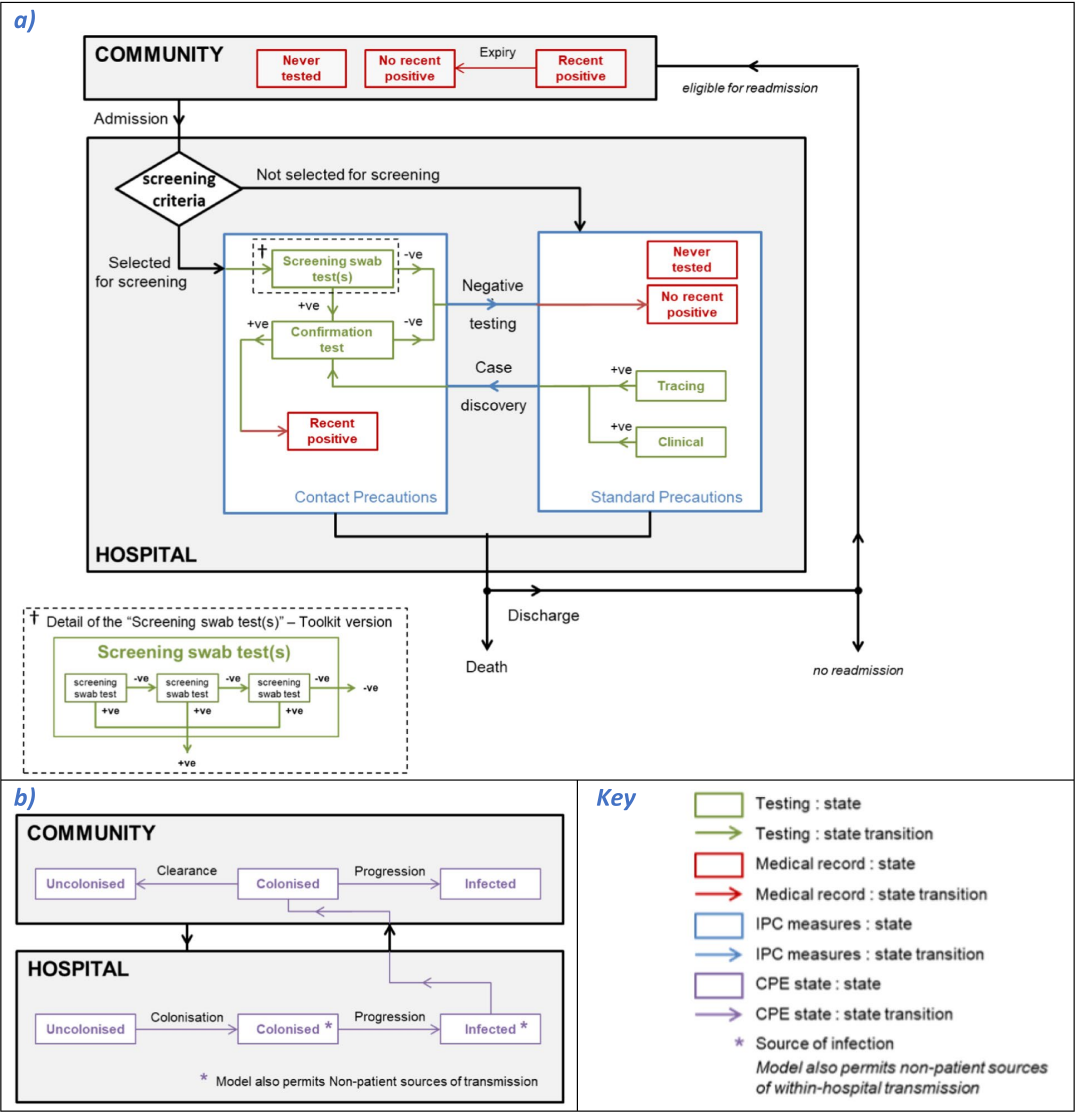
Model structure. Schematic depicting (**a**) CPE testing and (**b**) pathogen dynamics

An individual-based, stochastic mathematical model of an acute hospital and catchment population was developed. The model simulated CPE spread in individual patients admitted to the hospital for an overnight stay or longer. Each patient within the hospital could have one of three possible CPE states: (1) uncolonised, (2) colonised (including those with infections other than a bloodstream infection, BSI), or (3) infected (with a BSI, denoted separately to enable differential parameterisation for aspects such as mortality) (Fig. 1b). Those in either the colonised or infected state were assumed to be infectious and potential sources of CPE transmission whilst in hospital. Patients could transition between these states. For each time-step (daily), the probability that a susceptible patient became colonised was determined by the number of infectious patients on the same ward. Colonised patients could progress to an infected state.

Patients were admitted to the hospital in each of the three states; however, discharges could not occur whilst a patient was infected as these individuals still require treatment. Clearance of colonisation was assumed only to occur in the community. On admission, each patient’s medical record could show one of three states: a recent positive test for CPE (within 12 months), no recent positive test for CPE (no positive tests within last 12 months) or never tested for CPE. Beds in the hospital were assumed to be fully occupied at all times and allocated to homogenous wards, with no inter-ward transfers.

This hospital transmission model was used to simulate CPE screening and IPC recommendations from the 2016 ‘Acute trust toolkit for the early detection, management and control of carbapenemase-producing Enterobacteriaceae’ (hereafter referred to as the ‘Toolkit’ [10]) (Fig. 1a) and alternative screening protocols with modifications to admission screening criteria and screening test pathway (Table 1).

**Table 1:**
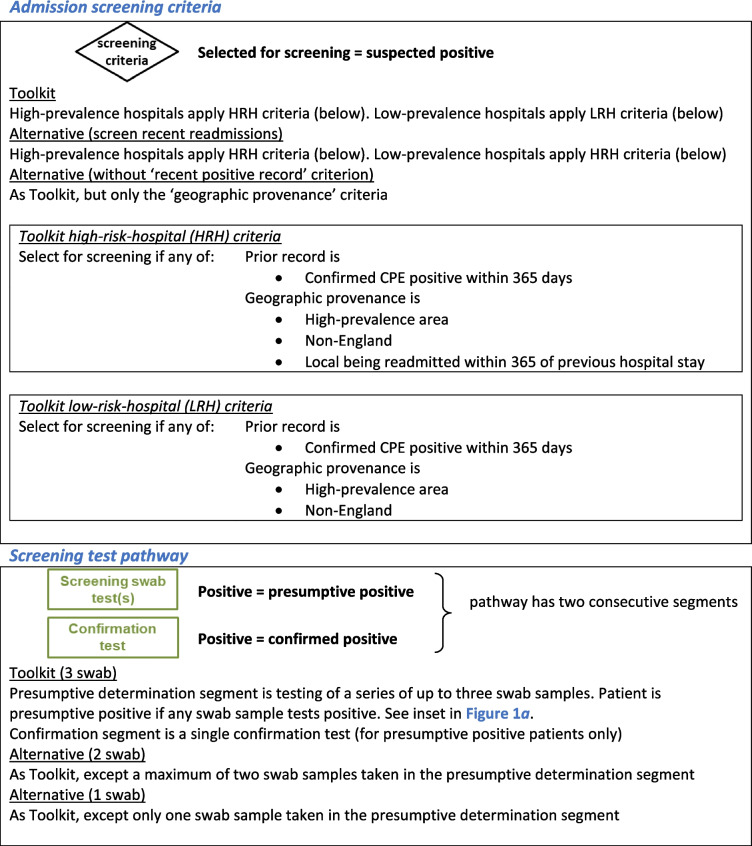
Screening protocols: admission screening criteria and screening test pathway

In the Toolkit, the selection criteria for screening on admission are recent (within 12 months) positive CPE tests or recent hospital stays in specified high-risk regions/abroad (i.e. settings identified as having a prior CPE problem). Contact precautions are pre-emptively applied to these patients during the screening process (which includes a presumptive determination before a confirmation test), as well as to confirmed positive patients.

In order to model these Toolkit screening selection criteria, provenance of admissions was simulated from four discrete geographies: (1) local (with prevalence-based risk determined by hospital location), (2) (non-local) low-prevalence areas, (3) (non-local) high-prevalence areas and (4) non-England. It was assumed that readmissions only occurred for local patients. At admission, a modelled patient was identified as a suspected positive and selected to undergo screening tests if they had a positive medical record or were admitted from a high-prevalence/non-England geography or were a local patient with a previous high-prevalence hospital stay within 365 days. Hence, modelled high-prevalence area hospitals included readmission (within 365 days) within their screening selection criteria (hereafter referred to as Toolkit high-risk-hospital (HRH) criteria), but those in low-prevalence areas did not (hereafter referred to as Toolkit low-risk-hospital (LRH) criteria). The alternative screening criteria evaluated was the application of the Toolkit HRH criteria in low-prevalence area hospitals (Table 1).

In simulations following the Toolkit screening test pathway, from the day of admission up to three screening samples (rectal swabs) were taken from suspected positive patients at intervals of 2 days (or after receipt of previous result, if longer) (Fig. 1a, inset). If all swab results were negative then the patient was re-categorised as suspected negative and their CPE medical record updated. If any swab returned a positive, the patient was categorised as a presumptive positive and a confirmation test undertaken. A negative confirmation test re-categorised the patient as suspected negative, whereas a positive confirmation result created a positive CPE medical record and triggered contact tracing. In contact tracing, patients on the same ward were swabbed (once), with a positive result triggering a presumptive positive label and the same confirmatory process. In addition, a random sample of patients outside the screening testing pathway were sampled (daily, and without replacement) for a clinically motivated test (assuming a blood culture sample), with a positive triggering a presumptive positive label and the same confirmatory process. The alternative screening test pathway evaluated was reducing the swabs taken from three to either two or only one (Table 1).

Results from all CPE testing (screening swabs, laboratory confirmation, contact tracing and clinically motivated testing) were retained as a medical record status for each patient, which may be ‘recent positive’ (which expires after 365 days), ‘no recent positive’ (tested but no CPE found, or expired positives only) or ‘never’ tested (Fig. 1a).

### Model parameterisation

Wherever possible, primary data were used to inform the model parameterisation (Table 2). Although CPE encompasses a variety of bacteria, antibiotics and resistance mechanisms, the evidence is currently not sufficiently rich to permit parameterisation for each of these separately. Hence, ‘CPE’ was modelled generically, and available data for any subset definition was used for parameterisation.

**Table 2:** Model parameters

**Process/Parameter**		**Value**				**Source**
**Hospital beds**^a^						
Number of beds in hospital		540				Informed by UK acute trust data [29]
**Ward beds**						
Number of beds on ward		60				Informed by UK study data [6]
**Number of admissions**^b^						
**Non-local patient**	Fixed maximum number of admissions	1				Assumption
**Local patient**	Probability of maximum admissions = $\alpha$ (given first admitted with *t* simulation days remaining)	Fitted values in Additional File 1 §1.a.ii				HES [30]
**Geographic source of patient**^b^ Proportion of admissions from each geography		**High-prevalence generic hospital**		**Low-prevalence generic hospital**		
**Local**	same region as hospital	0.969263		0.96897		Donker et al/HES [30, 31]
**Low-prevalence areas**	non-local low-prevalence regions (England)	0.006274		0.00812		Donker et al/HES [30, 31]
**High-prevalence areas**	non-local high-prevalence regions (England)	0.002251		0.000698		Donker et al/HES [30, 31]
**Non-England**	non-England geography	0.022212		0.022212		HES [30]
**Length of stay**^b^						
Duration of stay in hospital, distribution with median/IQR – given leaving by discharge		2 days / 1 – 5 days				HES [30]
Duration of stay in hospital, distribution with median/IQR – given leaving due to death		8 days / 3 – 18 days				HES [30]
Additional duration of stay if colonised (with or without CPE BSI)		7 days				Knight et al [12]
**Probability of death**						
Probability of leaving hospital due to death – without CPE BSI		2.9%				HES [30] all admission
Probability of leaving hospital due to death – with CPE BSI		48.4%				Xu et al/Budhgram et al/ Hauck et al [32–34]
**Colonisation on admission**^b^ Prevalence of CPE colonisation upon first admission		**High-prevalence generic hospital**		**Low-prevalence generic hospital**		HES/ERS/Donker et al/ European Antimicrobial Resistance Surveillance Network [30, 31, 35, 36]
**Local**	same region as hospital	0.000532		0.000042		
**Low-prevalence areas**	non-local low-prevalence regions (England)	0.000042		0.000042		
**High-prevalence areas**	non-local high-prevalence regions (England)	0.000532		0.000532		
**Non-England**	non-England geography	0.013		0.013		
**Infection on admission**						
Probability of patient colonised with CPE at admission presenting with a CPE BSI infection		310.88 per 100,000 admissions				PHE/HES [30, 37, 38]
**Force of infection** Transmission parameter $\beta ={\beta }_{0}+{\beta }_{1}({n}_{C}+{n}_{I})$		**From unknown sources** **(β**_**0**_**)**		**Per infectious patient on ward** **(β**_**1**_**)**		Estimated from the Bayesian Framework ward models using UK study data [6]
**Ward A **	Minimum	0.00001308		0.00008105		
	Median	0.00040719		0.00036252		
	Maximum	0.00188901		0.00075283		
**Ward B**	Minimum	0.00000698		0.00001354		
	Median	0.00023081		0.00024106		
	Maximum	0.00108142		0.00072196		
**Ward C**	Minimum	0.00000335		0.00002051		
	Median	0.00007870		0.00038651		
	Maximum	0.00035555		0.00148161		
**Progression rate**						
Probability of progression from CPE colonisation to CPE BSI (within hospital)		30.42 per 100,000 bed-days				PHE/ HES [30, 37, 38]
**Clearance (in the community) **						
Probability still CPE colonised, given readmitted after *n *days		$p\left(n\right)=\alpha +\left(1-\alpha \right){e}^{-\lambda n}$ $\alpha$ = 0.11724010, $\lambda$ = 0.04269721				Estimated from survival analysis of UK study data [6]
**Recent positive period**						
Duration of a Positive CPE status on patient’s record after discharge		365 days				Toolkit [10]
**Swab Test **						
Local CPE testing of rectal swabs from screening and from contact tracing sampling, test methodology used for parameterisation		Parameterised using data for MacConkey agar				Surveys of Acute trusts in England [3, 39]
**Confirmation test**						
Confirmation / reference lab testing of isolates for CPE, test methodology used for parameterisation		Parameterised using data for Cepheid Xpert Carba-R				Surveys of Acute trusts in England [3, 39]
**Clinical test**						
Local blood sample testing for carbapenem sensitivity, performed for clinical purposes, test methodology used for parameterisation		Parameterised using data for VITEK 2				Surveys of Acute trusts in England [3, 39]
**Test characteristics**		**Swab test**	**Confirmation test**		**Clinical test**	
Sensitivity		0.839	0.966		0.786	Meunier et al [40]
Specificity		0.917	0.986		0.820	Meunier et al [40]
Turnaround time (including to/from lab) – positive result		3 days	2 days		2 days	Expert opinion
Turnaround time (including to/from lab) – negative result		2 days	7 days		2 days	Expert opinion
**Clinical sample**						
Probability of selection for carbapenem sensitivity testing (per timestep), given suspected negative patient		1.58 per 1000 bed-days				Linked laboratory/ bed data from UK acute Trust [6]

^a^bespoke values estimated, from the same source, for individual trust verifications (values here are for generic high- and low-prevalence area hospitals)

^b^bespoke values calculated, from the same source, for referral region-typical hospitals and for individual trust verifications (values here are for generic high- and low-prevalence area hospitals)

#### Patient population

Patient populations were parameterised to model each of a generic low-prevalence area hospital and a generic high-prevalence area hospital. Additionally, we divided England into regions and parameterised a region-typical hospital model for each, using that region’s patient profile—these regions were the 14 ‘referral network regions’ that were identified by Donker et al. [31] from network analysis of patient movement data in England. For each of these 16 simulated hospitals, proportions of admissions by the four source geographies were estimated from England Hospital Episode Statistics (HES) allocated thus: ‘local’ (the hospital’s catchment area), ‘high-prevalence areas’ (the two regions with highest CPE prevalence in Donker et al. [31]), ‘low-prevalence areas’ (the remaining regions in England) and ‘non-England’ (non-England sources) (See Additional File 1 §1.a.i. for further details [30, 31, 37, 38].)

Patients’ movement parameters (source geography, length of stay, number of readmissions and time between admissions) were taken from analyses of all ordinary, overnight admissions to non-specialist acute hospital trusts in England (Hospital Episode Statistics, Admitted Patient Care [30]) from financial year 2013–2014 (FY1314, chosen to link with previously published patient network analysis [31, 41]). All admission spells (i.e. continuous stay in hospital) starting in FY 1314 (and completed before the end of FY1718) were used to determine the distribution of length of stay, with separate distributions for spells ending in discharge and those ending in death. These admission data (FY1314-FY1718) were also used to calculate distributions for the maximum number of readmissions for each patient (of local origin) across the 5-year modelled period, and for the length of the time between hospital spells (See Additional File 1 §1.a.ii. for further details [12, 30, 31].)

The probability of death for patients without CPE BSI was also calculated from the FY1314 HES cohort, whilst death probability for patients with CPE BSI was estimated from literature on observed in-hospital mortality from CPE BSI [32–34]. In the model, the expected length of stay and method of leaving (discharge or death) were randomly allocated at admission, adjusted for the patient’s CPE status, and were modified if the CPE status changed in-hospital via transmission or progression from colonised to infected.

As such, each generic and region-typical hospital used bespoke patient movement and local epidemiological parameters, estimated from empirical data from NHS Trusts in the corresponding locations.

#### CPE natural history

Linked admission and test data (antibiotic sensitivity and CPE screening) from a study [6] within a single UK acute hospital trust with endemic *bla*KPC-associated CPE transmission were used to estimate CPE transmission and clearance parameters: 4 years of data from across the trust (2013–2017), an 11-month subset (in 2015–2017) where additional bed-level data were available from a subset of wards (see Additional File 1 §1.b. for details [6, 42]).

To estimate the rate of transmission, a stochastic model of transmission was developed for each of three wards in the bed-level data-subset (Additional File 1 §1.b.ii [6, 43–47]) using a Bayesian framework: fitting patients’ observed CPE status, and estimating the unobserved transmission events, prevalence on ward admission and CPE test sensitivity. The rate at which a susceptible patient became colonised was assumed to be linearly dependent on the number of infectious patients and with constant pressure from any other transmission source, such as the environment [45–47]. Hence, the force of infection on an uncolonised patient on a ward with ${n}_{C}$ colonised patients and ${n}_{I}$ infected patients is given by $\beta ={\beta }_{0}+{\beta }_{1}({n}_{C}+{n}_{I})$ with ${\beta }_{0}$ and ${\beta }_{1}$ representing transmission from non-patient and patient sources, respectively. The analysis was also performed with $\beta ={\beta }_{1}({n}_{C}+{n}_{I})$, i.e. assuming no non-patient transmission sources (${\beta }_{0}$ fixed as zero). These Bayesian models were fitted in R by adjusting the code in Worby [43, 44] as available in the R package ‘bitrugs’ [48], using an uninformative prior. Median values from the posterior distributions of ${\beta }_{0}$ and ${\beta }_{1}$ were used in the calculation of the force of infection $\beta$ in the mathematical model.

To estimate the rate of clearance: a survival analysis, using the full hospital 4-year dataset, was conducted to estimate the distribution of time from hospital discharge until clearance (Additional File 1 §1.b.iii [6, 37, 38, 49–51]).

#### CPE infection prevention and control

For each test type (i.e. screening swab, confirmation and clinical), the sensitivity and specificity parameters corresponded to literature values associated with the modal method reported in a survey of 121 NHS England laboratories in 2018 [3] (see Additional File 1 §1.c.i [3, 39, 52, 53]). Total turnaround times were based on expert opinion assuming an uncomplicated investigation and no capacity ceiling.

The contact precautions recommended for suspected positives are a suite of measures such as hand hygiene, gloves and gowns and single rooms with en suite [10, 54]. This therefore will have a different overall effect on transmission depending on local practice, availability and adherence. The model presented here reflects overall effect of the IPC measures in the wards which generated the data used in the transmission parameter estimation.

### Model simulations and validations

CPE transmission was simulated over 5 years (with the first year a discarded burn-in period), and 300 simulation runs were completed for each combination of screening protocol and hospital. These 300 simulation runs included 100 runs for each of three parameterisations of the force of infection $\beta$ using the pairs of $\left\{{\beta }_{0},{\beta }_{1}\right\}$ coefficients. Each pair represented an alternative transmission setting (ward) and used the median fitted values from the corresponding Bayesian framework ward model above. Within each simulation run, parameter values defined by distributions were randomly drawn from the corresponding distribution. Parameter values and distributions (for high- and low-prevalence area hospital scenarios) are given in Table 2.

Model output was validated against 2018 CPE surveillance data [35] from five healthcare trusts (see Additional File 1 §3 [35]). Simulations using the limits of the (over-lapping) credible intervals from each of the three wards’ $\left\{{\beta }_{0},{\beta }_{1}\right\}$ estimations were used to investigate model sensitivity and, in model validation, to indicate a plausible range of variation (100 each pair, hence 300 additional simulations using the upper limits, 300 additional simulations using the lower limits, compared with the original 300 simulations using median values pairs). These simulations were also repeated using the estimates of the force of infection coefficients $\left\{{\beta }_{0},{\beta }_{1}\right\}$ obtained when the Bayesian Framework ward models were fitted under the alternative assumption that patients are the only possible transmission sources (${\beta }_{0}$ fixed as zero).

### Analyses of model output

Screening protocols, including both screening selection criteria and testing pathway, were evaluated in the generic high-prevalence and low-prevalence area hospital settings, and for the region-typical hospitals located in each of the 14 referral regions. Additionally, the generic low-prevalence area hospital was used as a template for a theoretical hospital which could experience a range of ‘local’ CPE prevalence values and could execute either the Toolkit LRH or Toolkit HRH screening selection criteria (Table 1).

Screening protocol evaluation includes estimations of the sensitivity and specificity of the admission selection criteria (i.e. comparing the categorisation according to the screening criteria vs patients’ true colonisation status at admission), and the performance of the two-segment screening test pathway, together with timeliness of results (measuring pathway performance with/without censoring due to discharge or death—events which are affected by the patient’s colonisation status). The combined ability of the screening criteria and testing pathway to identify colonised admissions was estimated.

## Results

### Performance of the admission screening criteria

In a generic low-prevalence area hospital, 43% (median, inter-quartile range [IQR] 32–55%) of CPE-colonised admissions were accurately categorised as suspect positive, and hence selected for screening tests, when applying the CPE Toolkit screening selection criteria (Table 1). In a generic high-prevalence hospital, the corresponding proportion was 54% (IQR 48–60%).

These criteria accurately categorised 99% (IQR 98–99%) of uncolonised admissions to a low-prevalence area hospital and 74% (IQR 74–74%) in a high-prevalence area hospital. So, given the underlying CPE prevalence, the majority of patients received a CPE suspected positive/negative status that matched their true CPE carriage status at admission in both low- and high-prevalence area hospitals (98% [IQR 98–99%] and 74% [IQR 74–74%] respectively). Although a higher proportion of colonised admissions met the selection criteria in the high-prevalence area hospital, the overall accuracy was lower due to the lower accuracy for uncolonised admissions, which still made up the majority of admissions.

The majority of suspected positive admissions under the Toolkit screening criteria met the geographic-based criteria (> 99%), and less than 1% had a positive CPE record (derived from testing in that same hospital). In the low-prevalence area hospital, admissions that met the screening criteria for geographical reasons were (by definition) non-local patients (constituting < 2% of admissions); conversely in the high-prevalence area, 94% (IQR 94–94%) of suspected positives were local patients returning within 12 months (with or without acquiring a positive CPE record in the previous visit(s)). An alternative set of screening criteria without a ‘recent positive record’ criterion (Table 1) did not affect the categorisation vs that under Toolkit criteria in the high-prevalence area hospital (as patients who met that criterion necessarily also met the local readmitted within 365 days criterion), but reduced the overall accuracy in the low-prevalence area hospital to 74% (IQR 74–74%) of all admissions.

**Fig. 2:**
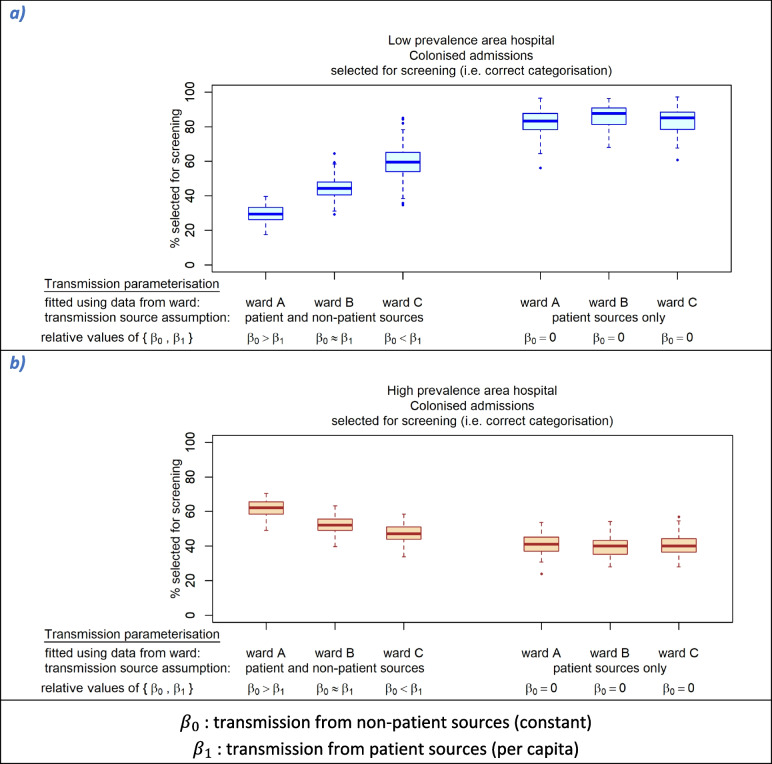
Accuracy of categorisation of colonised admissions by the Toolkit screening selection criteria: sensitivity to transmission parameterisation (by ward used for parameter estimation and transmission source assumptions). **a** Colonised admissions to generic low-prevalence area hospital. **b** Colonised admissions to generic high-prevalence area hospital

Colonised readmissions without a ‘positive’ record may include patients who were colonised by a transmission event during a previous hospital stay but whose colonisation was not discovered, and under Toolkit screening criteria these readmissions are only categorised as suspect positive (via the geographic provenance criteria) in a high-prevalence area hospital. The prevalence of such patients at readmission could be affected by underlying CPE prevalence and within-hospital transmission rates. Comparison of simulations using different transmission parameter estimates (from each of the wards used in parameter estimation, and with alternative parameters which had been estimated assuming no non-patient transmission sources, see Table 2 and Additional File 1 §2) showed that categorisation accuracy of colonised admissions under the Toolkit screening selection criteria was sensitive to patient and non-patient transmission rates (Fig. 2). In the low-prevalence area hospital, the proportion of colonised admissions selected for screening was lower in simulations using relatively low non-patient transmission parameters (vs the per capita patient source parameter), whereas the reverse relationship was seen in the high-prevalence area hospital.

Applying the Toolkit screening selection criteria to each of the referral region-typical hospitals (using the Toolkit HRH or LRH criteria (Table 1) according to the region’s prevalence) showed that for all the low-prevalence regions categorisation accuracy was broadly similar to that observed for the generic low-prevalence area hospital model (see Additional File 1 §4). In the region with the highest prevalence in England, accurate categorisation of colonised admissions for screening was 47% (IQR 42–55%) and the other region applying the HRH criteria (with second highest local prevalence in England) had the highest accuracy of colonised admission categorisation of all England regions (75%, IQR 68–79%).

**Fig. 3:**
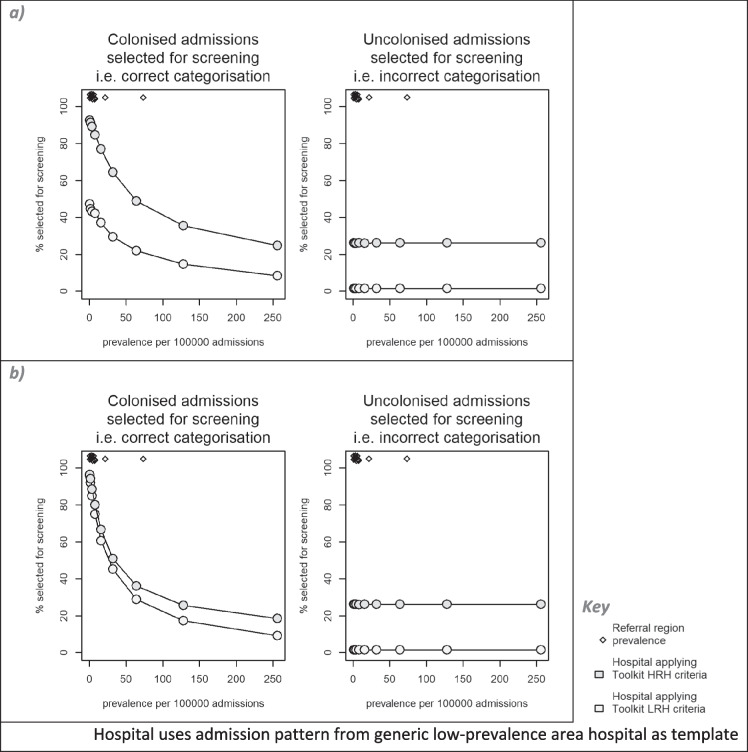
Accuracy of categorisation by admission screening criteria vs local CPE prevalence, under different transmission assumptions, for Toolkit and alternative criteria. Transmission parameterisation assumed **a** patient and non-patient transmission sources and **b** patient-only transmission sources

Low accuracy categorisation of both colonised and uncolonised admissions will cause undesirable outcomes, namely unknown and unprotected sources of CPE transmission, and unnecessary use of test and IPC resources, respectively. Using the theoretical hospital (with parameters as for the generic low-prevalence area hospital, except with variable local CPE prevalence) applying the Toolkit LRH screening selection criteria (Table 1) resulted in decreasing selection of colonised admissions for screening as the local CPE prevalence increased; the majority of colonised admissions were missed, and not selected for screening, for prevalence greater than ≈2 per 100,000 admissions (Fig. 3). Switching to applying the Toolkit HRH criteria (i.e. also selecting local recent readmissions) resulted in higher categorisation accuracy for colonised individuals; the accuracy of the Toolkit HRH criteria set similarly decreased with increasing prevalence, but the majority of colonised admissions were missed only when prevalence exceeded ≈64 per 100,000 admissions. For hospitals in the low-prevalence referral regions (prevalence 1–8 per 100,000), a switch from applying the Toolkit LRH criteria to the Toolkit HRH criteria doubled the proportion of colonised admissions which were selected for screening. The difference between the Toolkit LRH and HRH criteria was less when transmission was assumed to originate solely from patient sources, with the Toolkit LRH criteria (i.e. not testing local readmissions) generating a higher accuracy under this assumption compared with its application when non-patient sources were included. By contrast, the inaccurate categorisation of uncolonised admissions—hence patients undergoing unnecessary tests—was more stable across the same range of prevalence and was less affected by the choice of the screening selection criteria or transmission source assumptions.

### Performance of the screening test pathway

The first segment of the Toolkit screening test pathway is a series of up to three screening swab tests and the results of these swab test(s) determine if the patient is presumptive positive (Table 1, Fig. 1 inset). Under invariant circumstances (assuming constant CPE carriage status and no loss to follow-up, see Table 3), using multiple tests increased overall sensitivity vs a single test, but also decreased specificity (assuming tests are independent) (Table 3). Under invariant circumstances, the screening swab test(s) on suspected positives in the Toolkit pathway, to determine presumptive positive status, had a combined 99.6% sensitivity and 77.1% specificity. The screening test pathway is completed by the second segment: presumptive positives undergoing a (single) confirmation test, which has higher sensitivity and specificity than a single screening swab test. Overall, under invariant circumstances, combining presumptive and confirmatory testing from the Toolkit pathway delivered 96.2% sensitivity and 99.7% specificity. Curtailing the presumptive determination to a single-swab (1-swab alternative pathway Table 1) decreased the combined sensitivity to 81.0% (specificity 99.9%); however, it reduced the minimum negative result turnaround time (TAT) from 6 to 2 days (minimum positive TAT unchanged).

**Table 3:** Test performance of the screening test pathway segments, for Toolkit and alternative pathways

	**Invariant**^**a**^			**Dynamic**^**b**^					
				**Low-prevalence area**			**High-prevalence area**		
**Presumptive pathway**	**3 swabs** **(Toolkit)**	**2 swabs**	**1 swab**	**3 swabs** **(Toolkit)**	**2 swabs**	**1 swab**	**3 swabs** **(Toolkit)**	**2 swabs**	**1 swab**
**Presumptive determination test(s) outcome**									
**Sensitivity**	99.6	97.4	83.9	100.0	100.0	81.2	100.0	100.0	81.2
**Specificity**	77.1	84.1	91.7	72.4	83.2	93.4	72.4	83.5	93.3
**PPV**	-	-	-	6.6	8.3	11.8	0.9	1.1	16.0
**NPV**	-	-	-	100.0	100.0	99.8	100.0	100.0	100.0
**Confirmation test outcome** (all confirmation tests, including those prompted by clinical or contract tracing positive results)									
**Sensitivity**	96.6	96.6	96.6	100.0	100.0	100.0	100.0	100.0	100.0
**Specificity**	98.6	98.6	98.6	90.8	90.0	91.0	92.9	93.2	94.7
**PPV**	-	-	-	44.7	46.7	60.0	14.1	17.4	31.2
**NPV**	-	-	-	100.0	100.0	100.0	100.0	100.0	100.0

All values based on colonisation status at admission for constant denominator

^a^Invariant assumes: colonisation status does not change during stay, patients do not leave the hospital before determination complete

^b^Dynamic assumes: colonisation status may change during stay (status when each sample is taken is used in the stochastically simulated test result), patients may leave the hospital before determination from that pathway segment is complete (and removed from analysis sample)

**Fig. 4:**
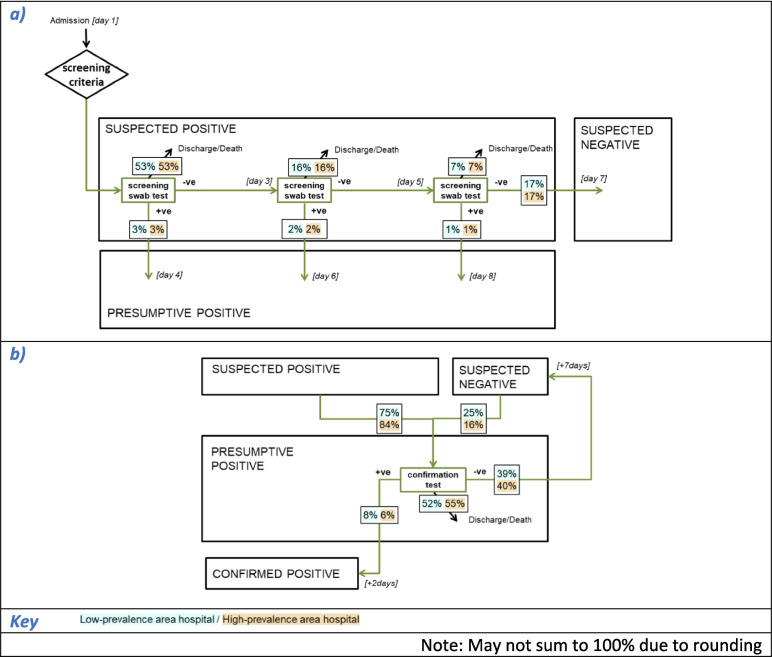
Proportion of admissions following each route through Toolkit screening test pathway, in generic low- and high-prevalence area hospitals. **a** Presumptive determination outcome for all screened admissions, denominator = total screened admissions. **b** Confirmation test outcome for all patients with samples sent for confirmation, denominator = total samples sent for confirmation

Calculating screening test pathway performance using the dynamic model simulations allowed for possible censoring due to discharge/death, which differs according to patients’ infection status, resulting in increased sensitivity and decreased specificity in results from tests completing later after admission, e.g. Toolkit presumptive determination specificity 72.4%, IQR 70.7–73.7% (Table 3). The flowchart in Fig. 4 shows the segments of the Toolkit screening testing pathway annotated to indicate the proportion of tested admissions who followed each possible route in simulations using the generic low-prevalence and high-prevalence hospitals (following screening selection using the Toolkit selection criteria). In both segments, for both hospitals, the majority were discharged before the screening pathway was complete, with 76% (IQR 76–77%, for both low- and high-prevalence area hospitals) leaving before the presumptive determination was completed. Thus, those patients had samples collected without generating a conclusive result.

Positive predictive values (PPV) were affected by prevalence within the screened cohort (dynamically determined by hospital admission and screening criteria) and were maximised for both presumptive and confirmation determinations when the screening test pathway was shortened to the 1-swab alternative (Table 1). Within the Toolkit recommendations, a confirmed positive record is used to initiate flagging the patient as requiring IPC should they be transferred to another facility; hence, the proportion of these flagged records which were due to false positives was minimised by the higher PPV under the 1-swab screening test pathway.

### Outcome from screening protocol (Screening Criteria and Screening Test Pathway combined)

**Fig. 5:**
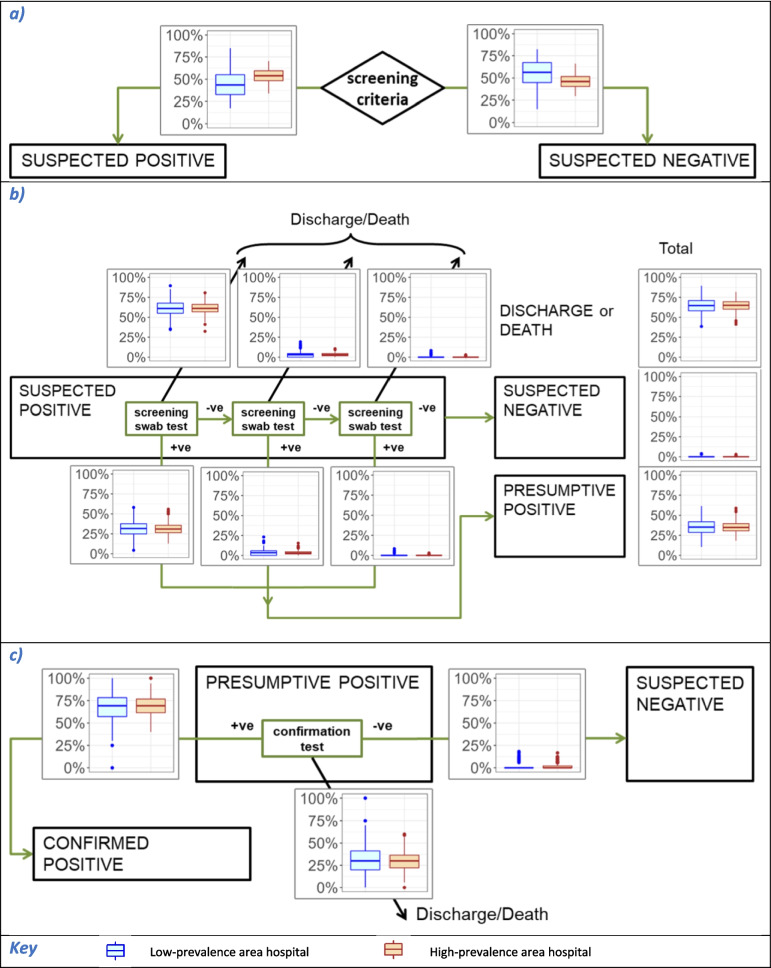
Route through each element of the Toolkit screening protocol for CPE-colonised admissions, low-prevalence area and high-prevalence area hospitals. Proportions following each possible route (for patients who were CPE-colonised at admission only). **a** Screening criteria: denominator = admissions. **b** Presumptive determination: denominator = screened admissions. **c** Confirmation test: denominator = samples sent for confirmation

Successful identification of a CPE-colonised patient via a screening protocol depends on the screening selection criteria and screening test pathway combining to conduct and deliver accurate test results in a timely manner. Applying the Toolkit protocol, 10% (IQR 7–13%) of CPE-colonised patients admitted to the generic low-prevalence area hospital were identified and 14% (IQR 11–17%) in the high-prevalence area hospital. The remainder were not identified (in that stay), due to either having not been selected for screening (low-prevalence area 57%, high-prevalence area 46%), or leaving hospital before presumptive determination or confirmation tests were completed (low-prevalence area 27 and 4%, high-prevalence area 35 and 6%, respectively) or due to receiving false-negative results (Fig. 5).

The overall identification results were sensitive to transmission parameter assumptions—assuming transmission was from patient sources only resulted in higher identification of CPE-colonised admissions for the low-prevalence area hospital (19%, IQR 15–25%) and lower identification for the high-prevalence hospital (10%, IQR 8–13%).

Changing the protocol to use the 1-swab alternative pathway, reducing the number of swabs taken for presumptive determination, resulted in the identification of 9% (IQR 6–13%) / 11% (IQR 9–15%) of CPE-colonised admissions in low- / high-prevalence area hospitals respectively but with fewer tests conducted (41% / 41% fewer screening swabs, 46% / 52% fewer confirmation tests in low- / high-prevalence area hospitals respectively). Using the theoretical variable-prevalence hospital, rates of identification decreased as prevalence increased, but at all prevalence levels more CPE-colonised patients were identified when the screening protocol changed to the Toolkit HRH screening criteria (including local readmission screening) than when using the Toolkit LRH criteria.

## Discussion

This mathematical model provides evidence to inform decisions on appropriate screening protocols to assist in the control of CPE within hospitals in England, by reporting colonisation detection under the programme outlined in the CPE Toolkit and alternatives (including those recommended for consideration by the Framework) when applied across the range of local CPE epidemiology observed in England (prevalence 1–74 per 100,000 admissions).

Case studies of improved nosocomial infection control via higher detection rates in admission screening [55, 56] highlight the desirability of screening criteria that effectively identify CPE-colonised patients. Under the Toolkit, the ability of screening criteria to select truly colonised patients on admission contributes not only to the performance of the screening programme, but also affects the potential for nosocomial transmission, as the categorisation is used to apply pre-emptive IPC measures to suspected positives. Our model showed that the Toolkit screening criteria applied in a low-prevalence area hospital accurately categorised the majority of admissions, but selected less than half CPE-colonised admissions for screening. Detection of CPE-colonised patients in low-prevalence area hospitals could be increased by including the screening of recent readmissions (up to 365 days); although only 20% of patients are readmitted within this timespan [30], these criteria could select over 80% of CPE-colonised admissions for screening.

A minority of CPE-colonised admissions were identified (and received a confirmed positive record) during their stay in both high- and low-prevalence area hospitals (14 and 10% respectively). This has implications beyond the IPC management of patients during that hospital stay; it also compromises the information that may be shared on discharge/transfer to determine appropriate IPC on leaving the hospital. Recorded confirmed CPE-colonisation potentially informs future screening decisions (readmissions to low-risk settings without a positive confirmation are not screened under the Toolkit protocol) and the extent to which diligent post-discharge test work-up can mitigate the future impact of CPE carriers who leave hospital before confirmation test results are received is limited by the proportions of unscreened patients. Confirmations are also used in surveillance data, so under-identification is a concern for understanding the magnitude of CPE prevalence in England. A point prevalence survey could confirm the scale of this issue, and inform future decisions on use of any screening criteria based on local prevalence.

Conversely, uncolonised patients form the majority of admissions (at all prevalence levels currently observed in regions in England) so their accurate identification has the larger effect on the IPC and laboratory resources required to deliver the screening pathway. In high-prevalence areas, the toolkit selection criteria have lower accuracy for uncolonised patients than in the low-prevalence areas. So, whilst IPC measures may also be beneficial against other pathogens, there is greater associated unnecessary recommended use of IPC measures, such as single rooms, which are limited and/or costly. These uncolonised patients incorrectly allocated for screening also undergo sample collection which may not be clinically necessary, and are not easily obtained, and so may not be conducive to a positive patient experience [57–59].

The impact on resources of wrongly selecting uncolonised patients for screening is further compounded by the reduced specificity through the multi-swab Toolkit screening test pathway generating more false positives (than a single-swab pathway), which require continued investigation with a confirmation test. Obtaining a negative result from a confirmation test has the longest turnaround time within this suite of tests; exacerbating the impact on IPC use for these (false positive) patients even if subsequently correctly confirmed as CPE-negative. This model indicates that switching to a single-swab pathway has almost no effect on carriage detection at admission but greatly reduces test usage (41% fewer screening swabs), freeing-up test capacity, which could be reallocated to broaden testing criteria or provide surveillance for long-stay patients [13]. The re-prioritisation of laboratory facilities during the SARS-CoV-2 pandemic [60], at the expense of other procedures including CPE admission screening, has emphasised the desirability of efficient screening protocols to optimise use of resources.

As well as the hospital’s prevalence context, our model indicates that within-hospital transmission affects the performance of the screening protocol (especially the contribution of readmitted patients with uncleared CPE colonisation gained during a previous stay). The handling of pathogen dynamics highlights a number of strengths of this model’s methodological approach. Firstly, both transmission and clearance parameters are directly estimated from longitudinal hospital prevalence data, via Bayesian inference and survival analysis respectively. Transmission and clearance processes are absent in some CPE screening models in the literature [12, 22, 61], or where they are present the estimates have been made indirectly using proxy measures [25] or model output validation is reliant on limited comparison datasets (single-point prevalence value [62, 63] or a single ward [21, 26]). Additionally, this model shows that the contribution of non-patient transmission sources impacts on the accuracy of selection of patients for screening, and to our knowledge, no screening model has considered non-patient-dependent transmission sources. Finally, this model uses stochastic dynamics which, compared with deterministic dynamics (adopted in some previous screening models [21, 25, 26]), is more suited to the simulation of small magnitude exposed populations—such as within a hospital ward—where random effects are more important to the dynamics.

The generalisability of results is limited by the single hospital source of the dataset used to estimate the transmission and clearance parameters and by its size (albeit a larger dataset than those used in previous models using single-ward validation); however, we have not found any other larger or similar datasets from other settings with which to broaden this evidence base. The source hospital is in a high-prevalence area and the predominant CPE mechanism (KPC) in this setting [6] is atypical for England as a whole [4] (although a major resistance mechanism globally [64]), but the range of estimates obtained from each of the source wards has permitted investigation of model sensitivity. The in-community clearance estimates are assumed to be independent of hospital and fully generalisable. Although the source hospital had a prior CPE problem, we note that ward C had a different cleaning history immediately prior to the study [6]—which may explain its lower β_0_ estimate compared with the other wards. Analysis of longitudinal data from more wards, from a wider range of epidemiological contexts (at hospital and ward scales), is required in order to fully understand which, if any, of the presented transmission scenarios is appropriate more generally or to inform setting-specific activities.

This study is limited by the paucity of species-specific, carbapenemase mechanism-specific or antibiotic-specific data for parameterisation. It has not been possible to generate sets of parameters for various subdivisions of the broad CPE definition. Again, further data is required to understand this situation, and whether the study data is an anomaly in this respect.

We could not find good quantitative data on the effect of IPC measures on CPE transmission, especially of the individual elements recommended in the Toolkit; hence, we assumed this was implicit in the estimated transmission parameters. Other settings may have different compliance or protocols, dictated by facilities as well as human adherence. Although the validation runs for other hospitals suggested this assumption was not unreasonable, this was only possible at an order of magnitude scale due to limited sample sizes. We also assume complete adherence to the application of screening criteria and sample collection; however, survey data indicates that this is likely to be an over-estimate in many settings [65]. Lower adherence will reduce the ability of the screening programme to identify CPE carriers compared with figures presented here, but may be mitigated if this non-adherence is non-random—for example if focussed towards short-stay patients given the high loss-to-discharge observed in the testing pathway. The model could be extended to incorporate either facility limitations [66] or quantified adherence, although would require better data on the effect on transmission of the corresponding IPC measure.

This model assumes homogeneity of susceptibility for all hospital patients, whereas epidemiological studies indicate risk factors such as specialist wards, comorbidity, patient demographics, antibiotic use and other factors result in heterogenous risks [67]. Specialist wards also influence patient movements associated with model assumptions on intra-hospital transfers and readmittance of non-local patients. This model could be extended to include such patient heterogeneities, subject to suitable data to permit parameterisation. Work to address these data gaps would enable the extension of the model to consider the performance of alternative screening criteria incorporating these factors; consideration of local and individual epidemiological factors is recommended within the 2020 Framework recommendations and is an inherently appealing direction for screening protocols.

Finally, we note that the model does not incorporate an economic analysis to include financial implications for the compared criteria strategy vs prevalence nor the confirmed positive identification vs test pathway. In addition to the morbidity and mortality associated with CPE infection [32], the financial costs associated with managing CPE outbreaks can be considerable [68–70]. We would expect decisions on strategy-switching to consider financial cost and morbidity measures in addition to the test-based data presented here.

## Conclusions

Using a novel modelling study—which, to the authors’ knowledge, is the only model in an English setting to consider the entire admission screening process (screening selection criteria and test pathway) incorporating the effects of within-hospital transmission dynamics—we have determined a number of potential changes to the 2016 Toolkit that could readily be initiated in English hospitals, which could improve the detection of cases and reduce the clinical and opportunity costs of screening. These include switching to a faster pathway of a single-swab test (rather than multiple tests over a period of days) and increasing colonisation detection by expanding admission screening criteria to consider recent past admissions to all hospitals (including readmissions to hospitals in ‘low-risk areas’). The Framework protocol has a default one-swab pathway and being an inpatient in any hospital (within a year) is considered a risk factor in the screening selection decision. Additionally, we have identified some critical data gaps that, if addressed, could improve the model in the future—including a CPE point prevalence study, adherence to IPC measures and testing recommendations. Further work on determining the nature of transmission sources (e.g. environment or other patients) would assist settings in choosing an appropriate screening programme as part of their activities to restrict nosocomial CPE transmission.

### Supplementary Information

**Additional file 1: Supplemental information: §1.** Parameterisation (§1.a. Patient population and movements: §1.a.i. Patient source geography and associated prevalence on admission, §1.a.ii. Length of Stay, Maximum number of admissions per patient, Time between admissions; §1.b. CPE natural history : §1.b.i. Definitions used in test data analysis for CPE natural history parameters, §1.b.ii. Transmission estimation, §1.b.iii. Clearance estimation; §1.c. CPE infection prevention and control : §1.c.i Screening, contact tracing and clinical tests sensitivity and specificity), §2 Additional parameters, §3 Model validation, §4 Simulation results for region-typical hospitals.

## Data Availability

Hospital Episode Statistics (HES) data are available from NHS Digital, but restrictions apply to the availability of these data, which were used under licence for this study, and so are not publicly available. HES data are however available from the authors upon reasonable request and with permission of NHS Digital. The empirical hospital data analysed during the current study (linked admission and test data from an acute hospital trust) are available from the TRACE Study Group, but restrictions apply to the availability of these data, which were used with permission for the current study, and so are not publicly available. Data are however available upon reasonable request and with permission of TRACE Study Group.

## Abbreviations

BSI: Bloodstream infection
CPE: Carbapenemase-producing Enterobacterales
ERS: Electronic Reporting System
Framework: Framework of actions to contain carbapenemase-producing Enterobacterales [11]
FY: Financial year
HES: Hospital Episode Statistics
IPC: Infection prevention and control
IQR: Inter-quartile range
KPC: *Klebsiella pneumoniae* Carbapenemase
NHS: National Health Service
NPV: Negative predictive value
PHE: Public Health England
PPV: Positive predictive value
TAT: Turnaround time
Toolkit: Acute trust toolkit for the early detection, management and control of carbapenemase-producing Enterobacteriaceae [10]
Toolkit HRH criteria: Toolkit admission screening criteria for high-risk hospital (modelled as in Table 1)
Toolkit LRH criteria: Toolkit admission screening criteria for low-risk hospital (modelled as in Table 1)
UK: United Kingdom
