# Supplementary material for: Model-based evaluation of admission screening strategies for the detection and control of carbapenemase-producing Enterobacterales in the English hospital setting
Source: BMC Med. 2023 Dec 12;21:492. doi: 10.1186/s12916-023-03007-1 (PMC10717398; doi:10.1186/s12916-023-03007-1)
Supplement: Supplementary file 1 — Additional file 1: Supplemental information: §1. Parameterisation (§1.a. Patient population and movements: §1.a.i. Patient source geography and associated prevalence on admission, §1.a.ii. Length of Stay, Maximum number of admissions per patient, Time between admissions; §1.b. CPE natural history : §1.b.i. Definitions used in test data analysis for CPE natural history parameters, §1.b.ii. Transmission estimation, §1.b.iii. Clearance estimation; §1.c. CPE infection prevention and control : §1.c.i Screening, contact tracing and clinical tests sensitivity and specificity), §2 Additional parameters, §3 Model validation, §4 Simulation results for region-typical hospitals. [file 12916_2023_3007_MOESM1_ESM.docx]

**Model-based evaluation of admission screening strategies for the detection and control of carbapenemase-producing Enterobacterales in the English hospital setting**

**ADDITIONAL FILE 1**

Contents

[1. Parameterisation 2](#_Toc98165953)

[a. Patient population and movements 2](#_Toc98165954)

[i. Patient source geography and associated prevalence on admission 2](#_Toc98165955)

[ii. Length of Stay, Maximum number of admissions per patient, Time between admissions 3](#_Toc98165956)

[b. CPE natural history 5](#_Toc98165957)

[i. Definitions used in test data analysis for CPE natural history parameters 5](#_Toc98165958)

[ii. Transmission estimation 6](#_Toc98165959)

[iii. Clearance estimation 8](#_Toc98165960)

[c. CPE infection prevention and control 9](#_Toc98165961)

[i. Screening, contact tracing and clinical tests sensitivity and specificity 9](#_Toc98165962)

[2. Additional parameters 10](#_Toc98165963)

[3. Model validation 10](#_Toc98165964)

[4. Simulation results for region-typical hospitals 10](#_Toc98165965)

# Parameterisation

## Patient population and movements

### Patient source geography and associated prevalence on admission

Each modelled hospital (generic low-prevalence area, generic high-prevalence area and typical hospitals for each referral-region) had an associated set of admission geography profiles (proportions for the local area, non-local low-prevalence areas, non-local high-prevalence areas, and non-England sources), which were estimated using reported admissions in the HES APC FY1314 cohort^30^. The non-England proportion used a nationally reported figure which was applied to all modelled hospitals – and in the application of Toolkit screening selection criteria the non-England origin was considered high-risk. The subdivisions of England admissions (local, high-prevalence areas, low‑prevalence areas) utilised the referral regions calculated in Donker et al^31^ (**Additional Table 1**). Regions 4 and 5 (the two highest prevalence regions) were considered high-prevalence in the Toolkit screening selection criteria (***Table 1*** in the manuscript), all others low-prevalence. For each region-typical hospital, the admission profile was calculated from the regional England admissions data from Donker et al’s analysis. The generic low‑prevalence area hospital used the proportions from the sum of low-prevalence regions admissions, and similarly for the generic high-prevalence hospital.

Additional Table 1. Referral regions

| Number | Name in Donker et al^31^ | Number of trusts | Proportion of all England admissions^†^ | Prevalence-based risk assumption |
| --- | --- | --- | --- | --- |
| 1 | Sheffield | 5 | 3.6% | Low |
| 2 | Leeds | 9 | 7.4% | Low |
| 3 | Newcastle | 9 | 6.3% | Low |
| 4 | Liverpool | 7 | 4.3% | High |
| 5 | Manchester | 9 | 6.7% | High |
| 6 | Lancashire | 4 | 3.6% | Low |
| 7 | Oxford | 5 | 3.9% | Low |
| 8 | Leicester | 8 | 6.6% | Low |
| 9 | Birmingham | 15 | 11.7% | Low |
| 10 | Southampton | 8 | 4.9% | Low |
| 11 | Bristol | 12 | 7.5% | Low |
| 12 | Cambridge | 8 | 5.3% | Low |
| 13 | London South & West | 18 | 13.2% | Low |
| 14 | London North | 23 | 14.9% | Low |

^†^ may not sum to 100% due to rounding (admissions from HES APC FY1314^30^)

For each hospital’s four patient source geographies, a CPE prevalence on admission was estimated. Prevalence for overseas origin used a value for Europe. Prevalence on admission for each England referral region was assumed to be the prevalence reported in Donker et al^31^ for that region, and these values used for local admissions. Admission-weighted averages of the constituent regions’ prevalence values were used to estimate values for low-prevalence and high-prevalence origins. A proportion (constant across all geographies) of colonised admissions who presented with CPE BSI was estimated from the community-acquired CPE BSI reported in surveillance data^37, 38^.

### Length of Stay, Maximum number of admissions per patient, Time between admissions

Each modelled hospital had associated distributions for length of stay (for stays ended by death), length of stay (for stays ended by discharge), for number of admissions per individual patient within the model period, and for the time between consecutive hospital stays, from which randomly-drawn values were used to determine the timing of a patient’s movements in/out of hospital. These distributions were taken from analyses of all ordinary, overnight admissions to non-specialist acute hospital trusts in England (Hospital Episode Statistics, Admitted Patient Care (HES APC))^30^, using a cohort of all admissions from financial year 2013-14 (FY1314) (excluding Mid Staffordshire NHS Foundation Trust, which was disbanded during this period). Distributions were calculated for each simulated hospital, with using collated admissions from all trusts in England (used for the generic low-prevalence area and generic high‑prevalence area hospitals), or the trusts within the referral region^31^ for the region-typical hospitals.

Length of stay distributions were calculated separately for spells which ended with the patient’s death or with patient’s discharge. At admission, a Bernouilli trial determined if the patient would leave by discharge or death (with a different probability of death for those with BSI), and the patient’s expected length of stay was drawn randomly from a distribution corresponding to the patient’s anticipated reason for leaving and CPE colonisation status. The distributions used for uncolonised patients were taken directly from proportions of each duration observed for the HES APC FY1314 cohort (**Additional Figure 1**). These distributions were adjusted by an estimate of the mean additional length of stay for CPE carriers (estimated from empirical data from an England trust^12^) to generate the length of stay distributions for patients with CPE (including those in an infected state). If a transmission event occurred in hospital, the patient’s length of stay was extended by the fixed additional stay value for colonised patients (as above). If a patient progressed to the infected (BSI) state whilst in hospital, the anticipated reason for exit was redrawn and, if necessary, the corresponding anticipated time of death was calculated.

Additional Figure 1. Distribution - Length of stay (generic settings, uncolonised patients)

| 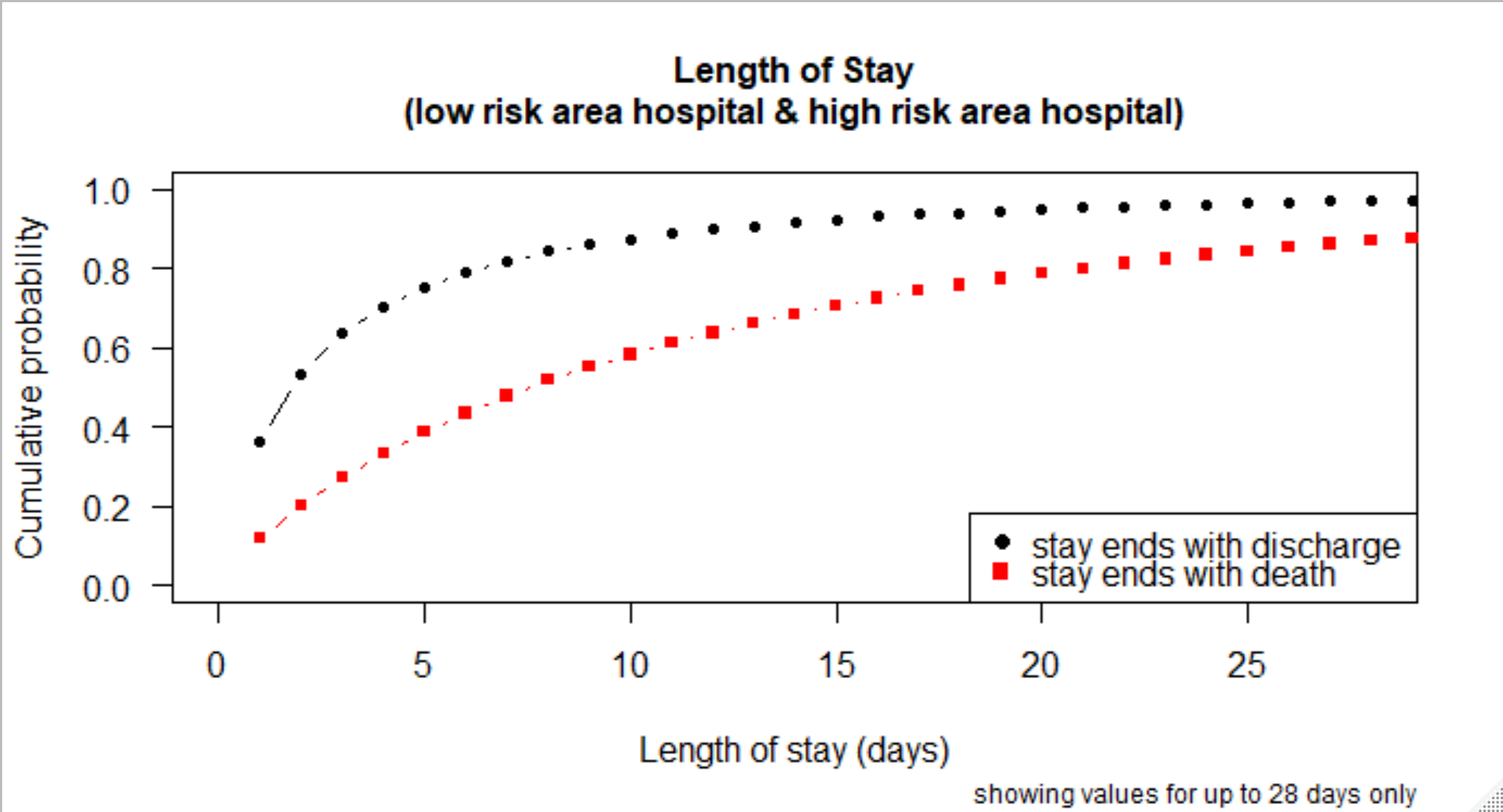 |
| --- |

A maximum possible number of admissions 𝑎_𝑚𝑎𝑥_ to the hospital within the 5-year period of the model simulation was allocated to each patient on their first admission. For non-local patients this was fixed at 1. For local patients this was randomly selected from a distribution which was estimated from HES APC and was a function of the day of their first admission (those with later first admissions having lower maxima, as shorter remaining simulation time).

To estimate these distributions, the number of admissions to the same trust within 183, 365, 730, 1095, 1460 and 1825 days were obtained for patients with admission in the HES APC FY1314 cohort, as used above. Where trusts were merged before the end of FY1718, this merger was backdated to the start of the cohort period. The proportion of patients in the cohort with each admission count was calculated for each of these windows, and then separate functions of the form

$$f_{a}\left( t \right)=\mu_{a}\ln t+\gamma_{a}$$

were fitted (using least squares) to the set of proportions for each of 𝑡 ∊ {183, 365, 730, 1095, 1460, 1825 days} for each value of admission count (𝑎). Fitted values of {$\mu_{a}$,$\gamma_{a}$} are given in **Additional Figure 2a**.

This set of functions $\{f_{a}\}$, with floor 0 and ceiling 1 applied, were used to generate the probability that a patient, first admitted with *t* simulation days remaining, was allocated 𝑎_𝑚𝑎𝑥_ = 𝑎. Examples of these fitted curves (for proportion of patients with 1, 2, 3, 4, 5 or 6 as maximum number of admissions) for the generic settings are shown in **Additional Figure 2b**.

Additional Figure 2. Distribution – Maximum possible number of admissions, 𝑎 (generic settings)

| ***a)***  $f_{a}\left( t \right)=\mu_{a}\ln t+\gamma_{a}$   \| \| 𝑎 \| 𝜇_𝛼_ \| 𝛾_𝛼_ \| \| --- \| --- \| --- \| \| 1 \| -0.13565 \| 1.568846 \| \| 2 \| 0.041154 \| -0.09764 \| \| 3 \| 0.032194 \| -0.14416 \| \| 4 \| 0.020517 \| -0.10235 \| \| 5 \| 0.013005 \| -0.0677 \| \| 6 \| 0.008478 \| -0.04525 \| \| 7 \| 0.005639 \| -0.0305 \| \| 8 \| 0.003756 \| -0.02044 \| \| 9 \| 0.002629 \| -0.0144 \| \| 10 \| 0.001837 \| -0.01009 \| \| 11 \| 0.001481 \| -0.00832 \| \| 12 \| 0.001022 \| -0.00568 \| \| 13 \| 0.00069 \| -0.0038 \| \| 14 \| 0.000574 \| -0.00322 \| \| 15 \| 0.000436 \| -0.00239 \| \| 16 \| 0.000333 \| -0.00185 \| \| 17 \| 0.000338 \| -0.00195 \| \| 18 \| 0.000182 \| -0.00099 \| \| 19 \| 0.000179 \| -0.00104 \| \| 20 \| 0.0000362 \| 0.000477 \| \| \| --- \| --- \| --- \| --- \| --- \| --- \| --- \| --- \| --- \| --- \| --- \| --- \| --- \| --- \| --- \| --- \| --- \| --- \| --- \| --- \| --- \| --- \| --- \| --- \| --- \| --- \| --- \| --- \| --- \| --- \| --- \| --- \| --- \| --- \| --- \| --- \| --- \| --- \| --- \| --- \| --- \| --- \| --- \| --- \| --- \| --- \| --- \| --- \| --- \| --- \| --- \| --- \| --- \| --- \| --- \| --- \| --- \| --- \| --- \| --- \| --- \| --- \| --- \| --- \| | ***b)***  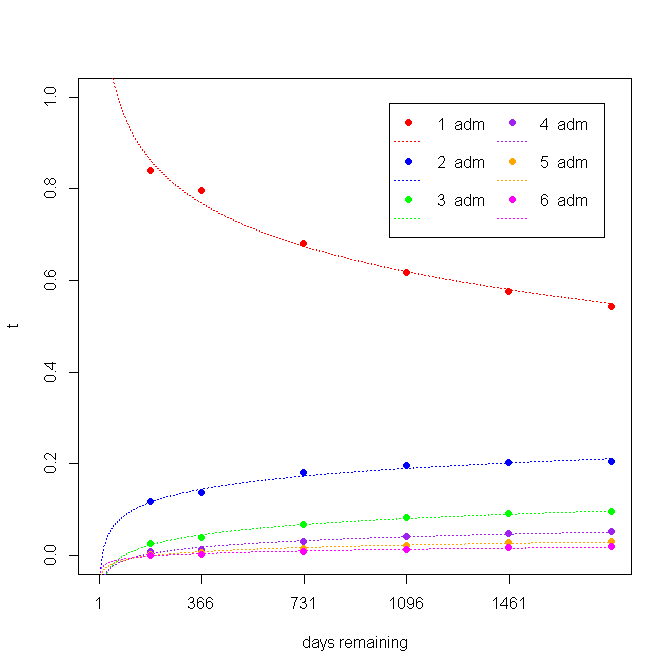 |
| --- | --- | --- | --- | --- | --- | --- | --- | --- | --- | --- | --- | --- | --- | --- | --- | --- | --- | --- | --- | --- | --- | --- | --- | --- | --- | --- | --- | --- | --- | --- | --- | --- | --- | --- | --- | --- | --- | --- | --- | --- | --- | --- | --- | --- | --- | --- | --- | --- | --- | --- | --- | --- | --- | --- | --- | --- | --- | --- | --- | --- | --- | --- | --- | --- | --- |

***a) probability function coefficients. b) function fitting examples for a = 1, 2, 3, 4, 5 and 6***

Patients exiting the hospital for the final time (either via death or having reached 𝑎_𝑚𝑎𝑥_ admissions) are replaced in the population, otherwise they are scheduled for readmission after a given number of days which was randomly drawn from a distribution of estimated time until readmission. This distribution was estimated from the HES APC FY 1314 cohort of admissions, measuring the time until their next admission spell to the same trust, considering spells completed before the end of FY1718 (**Additional Figure 3**). Where trusts were merged before the end of FY1718, this merger was backdated to the start of the cohort period. Patients with a single stay within the period FY1314 - FY1718 (i.e. no readmission after the initial cohort-defining stay) were censored, to provide normalised distributions.

Additional Figure 3. Distribution – time until readmission (generic settings)

| 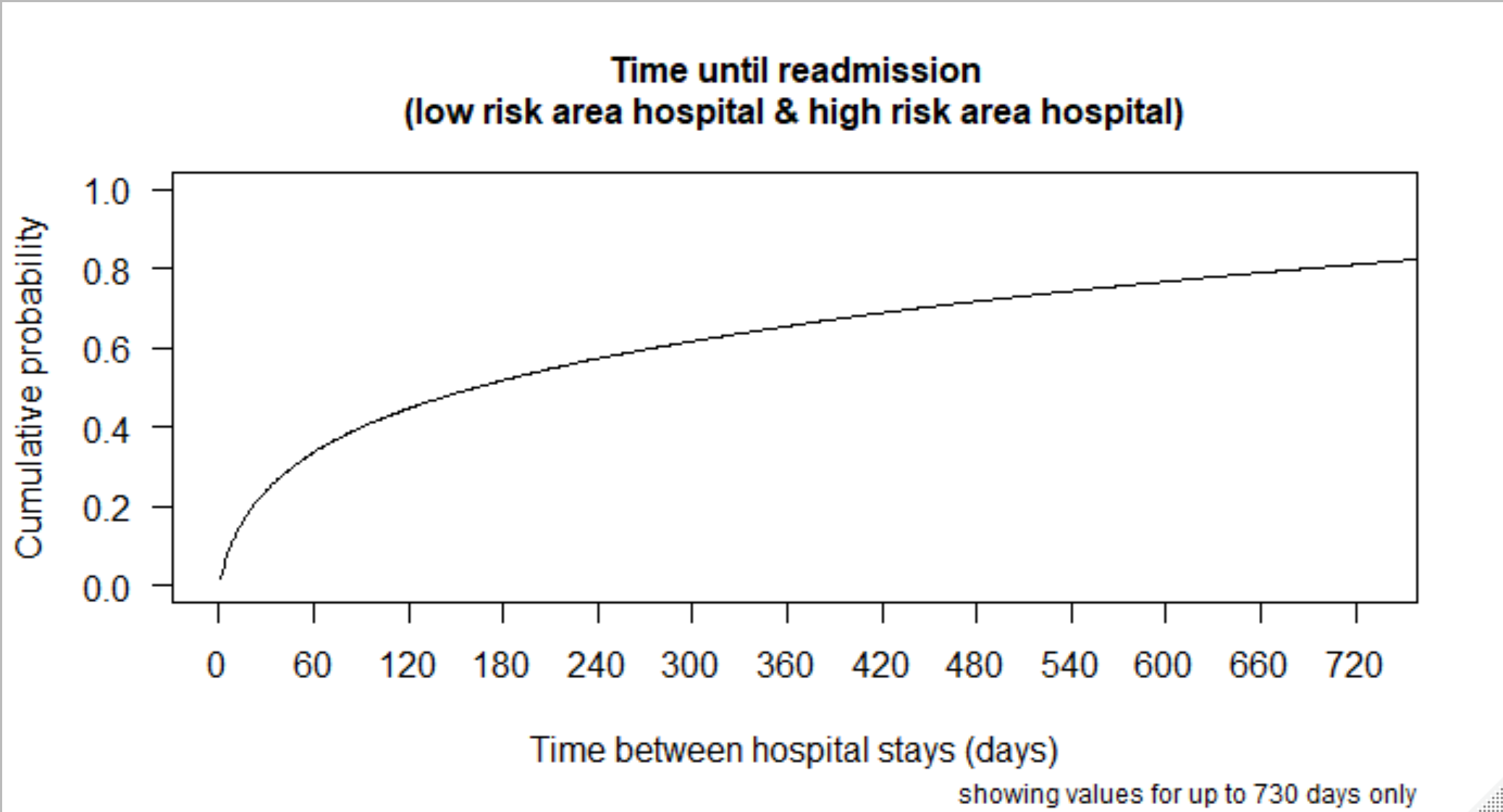 |
| --- |

## CPE natural history

The parameterisation of transmission and clearance processes used data from linked hospital stay and test databases from an acute hospital trust in England^6^. Test results were used to uniquely infer a patient’s CPE colonisation status on the date when the test sample was collected. (Colonised individuals with CPE BSI were not treated separately in these analyses.) When multiple tests were conducted or multiple samples collected on a given date, then the patient was inferred to be CPE colonised on that date if any test had a positive result (an uncolonised inference was made only when all results were negative). The patient’s status on other dates was unknown and inferred as part of the parameter estimation processes described below (transmission and clearance).

### Definitions used in test data analysis for CPE natural history parameters

Two definitions of test inclusion (**Additional Table 2**) were considered for use during these CPE natural history parameterisation analyses. The ‘CRE-based’ definition used was based on resistance to meropenem or imipenem as resistance to these carbapenems offers an acceptable indication of carbapenemase presence^42^. The “CP-based’ definition considered additionally including tests using PCR to identify carbapenemase producers, and a positive from either test inferred a positive result.

However, unsatisfactory results were obtained when using the CP-based definition of a CPE test during both transmission parameterisation (examining inferred test sensitivity, see §1.b.ii) and clearance estimation (see §1.b.iii). Furthermore, as a more homogeneous definition the CRE-based definition offered potentially better between-test stability, beneficial in these longitudinal analyses of colonisation status. Hence the CP‑based definition was rejected, and parameters estimated using the CRE‑based test definition were used in the hospital model.

Additional Table 2. CPE status inference test definition

|  | | Test inclusion | Positive result |
| --- | --- | --- | --- |
| CRE-based definition  *(used in hospital model)* | | Samples (of any type and site) from which Enterobacteriales colonies were formed and which were tested for susceptibility to meropenem or to imipenem | Resistant result, for either antibiotic, implies positive for CPE |
| CP-based definition  *(rejected)* |  | Samples (of any type and site) from which Enterobacteriales colonies were formed and which were tested for susceptibility to meropenem or to imipenem | Resistant result, for either antibiotic, implies positive for CPE |
|  |  | Samples which were examined using PCR methods for carbapenemase producers, carbapenemase positive samples were then cultured ^†^ | Enterobacteriales present implies positive for CPE |

^†^ there were multiple changes to the exact test protocols and methodology across the full dataset period.

### Transmission estimation

The transmission parameters were estimated from longitudinal data linking bed-level hospital movement and test data from an acute hospital trust in England^6^. Transmission was assumed to only occur within a “ward”, where wards are defined by shared staff/facilities (i.e. potential transmission sources), not by a room name. Under this definition, the six differently-named spaces with bed-level data formed three different spaces from the perspective of enterobacteriales transmission. Each of these three wards was analysed separately, making no assumptions about homogeneity across wards. This ward definition was also used to estimate the ward size used in the hospital model. More than one speciality was represented in the wards in the data, including an acute medical admissions unit, a cardiac unit (including surgical patients), and a geratology unit. The dates analysed within the linked dataset optimised availability of complete bed-occupancy data for each ward.

A Bayesian Framework model was used to estimate parameters for the force of infection (within a ward) by fitting patients' observed CPE status (known for the days when CPE-tested samples were collected), and estimating the unobserved transmission events, prevalence on ward admission and CPE test sensitivity (for each of three ward datasets). The modelling was conducted in R using code adapted from that used in Worby et al^43, 44^. All individuals who did not have a test throughout the study and those who only had negative tests were assumed to have remained susceptible during their stay on the ward, otherwise unobserved daily CPE status values were permitted to vary as part of the model fitting.

Transmission pressure was assumed to be linearly dependent on the total number of patients who were CPE positive and with constant pressure from any other transmission source, such as the environment^45-47^. Infected (BSI) and colonised patients were assumed to contribute to transmission dynamics identically, as they were not distinguished in the CPE status inference. Hence, the force of infection 𝛽 on a ward with 𝑛_𝐶_ colonised patients and 𝑛_𝐼_ infection patients is given by

$$\beta=\beta_{0}+\beta_{1}(n_{C}+n_{I})$$

where 𝛽_0_ : transmission coefficient from non-patient sources

𝛽_1_ : transmission coefficient from patient sources

The analysis was also performed assuming no non-patient transmission source, i.e. with fixed 𝛽_0_=0 (see **section 2 ‘Additional parameters’**). Marginal distributions for the fitted transmission parameter estimates are shown in **Additional Figure 4a**, the median values for each ward dataset were used in the hospital model. We note that fitting the model to data from ward C is based on fewer inferred transmission events than either ward A or B (by an order of magnitude).

In addition to the transmission parameters for use in the hospital model, this analysis simultaneously fitted estimates for the admission prevalence and test sensitivity. Values taken from direct observation were preferred for these parameters in the main model, but the sensitivity values obtained from this Bayesian transmission parameter model provided evidence to validate the transmission parameter estimation. Fitted test sensitivity values were compared with individual test empirical values for a range of methodologies (78.6%, 83.9%, 96.6% for the three CPE test methodologies used in the hospital model). Estimates from each ward were plausible using the ‘CRE-based’ test definition, but less plausible with the rejected ‘CP-based’ test definition (**Additional Figure 4b**).

Additional Figure 4. Transmission parameter estimation

| ***a)***   \|  \| Assume patient (𝛽_1_) and non-patient (𝛽_0_) transmission sources \| \| Assume patient-only sources \| \| --- \| --- \| --- \| --- \| \|  \| 𝛽_0_ \| 𝛽_1_ \| 𝛽_1_ \| \| Ward A \| 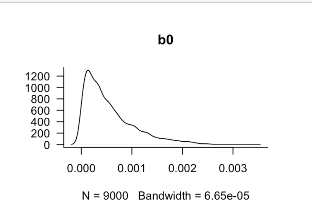 \| 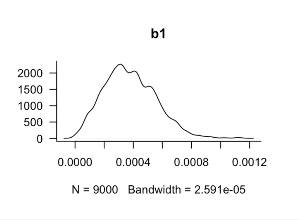 \| 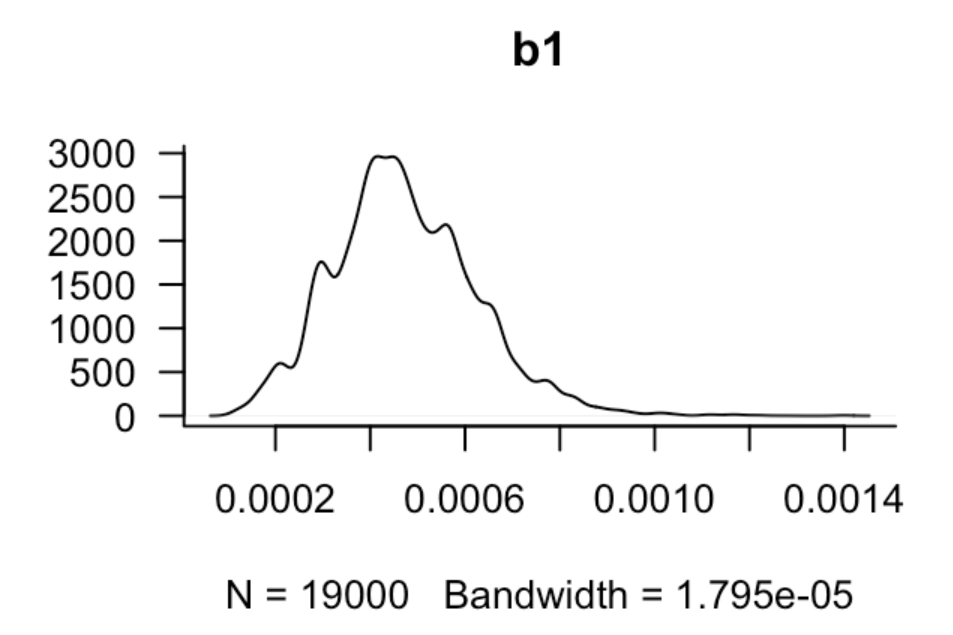 \| \| Ward B \| 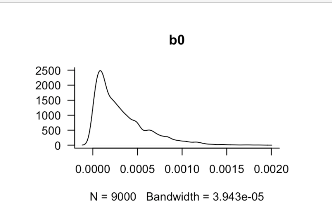 \| 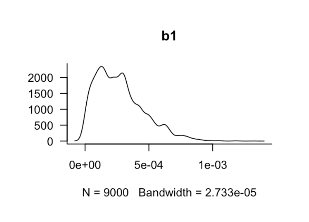 \| 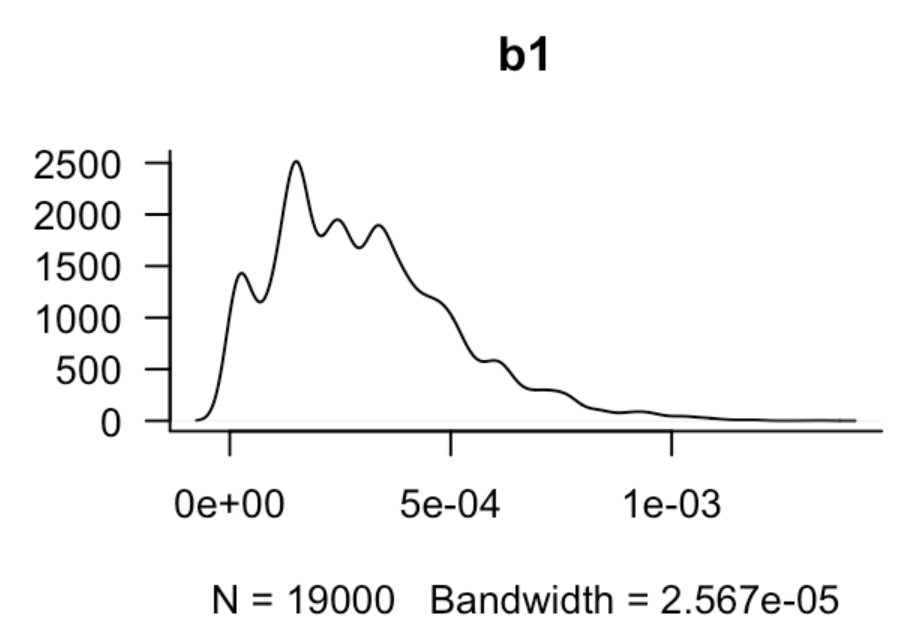 \| \| Ward C \| 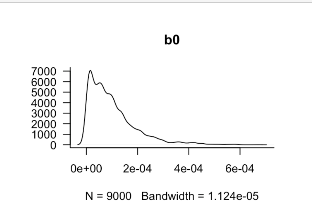 \| 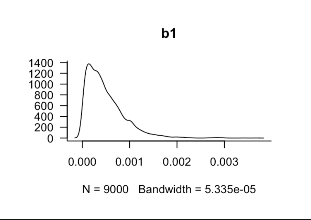 \| 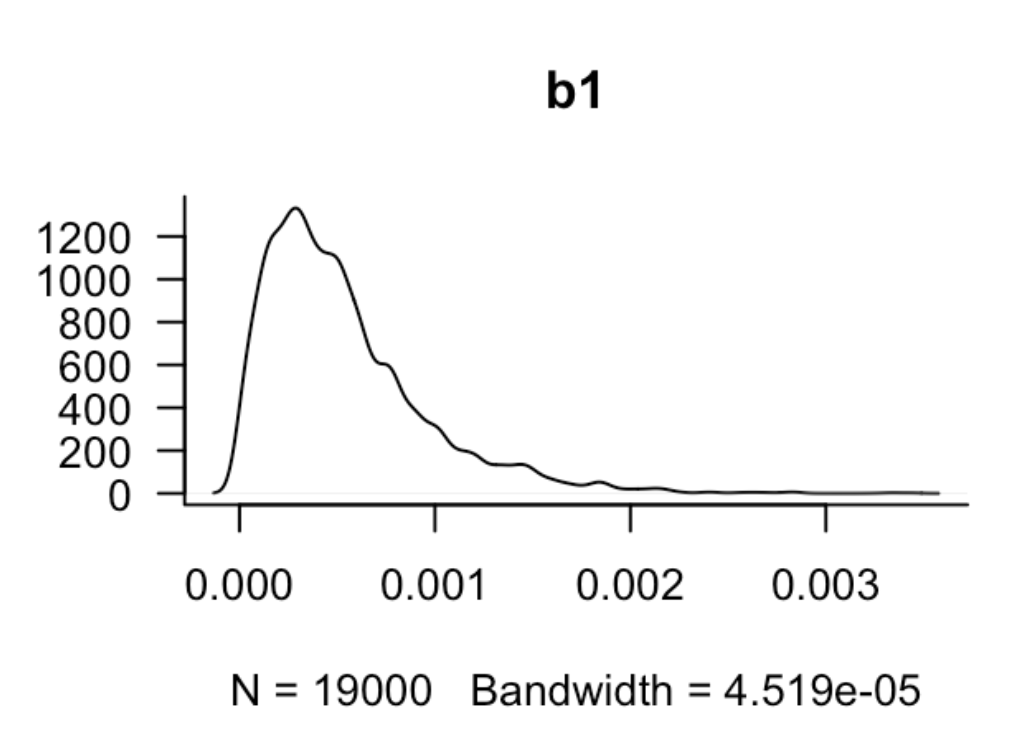 \| |
| --- | --- | --- | --- | --- | --- | --- | --- | --- | --- | --- | --- | --- | --- | --- | --- | --- | --- | --- | --- | --- |
| ***b)***  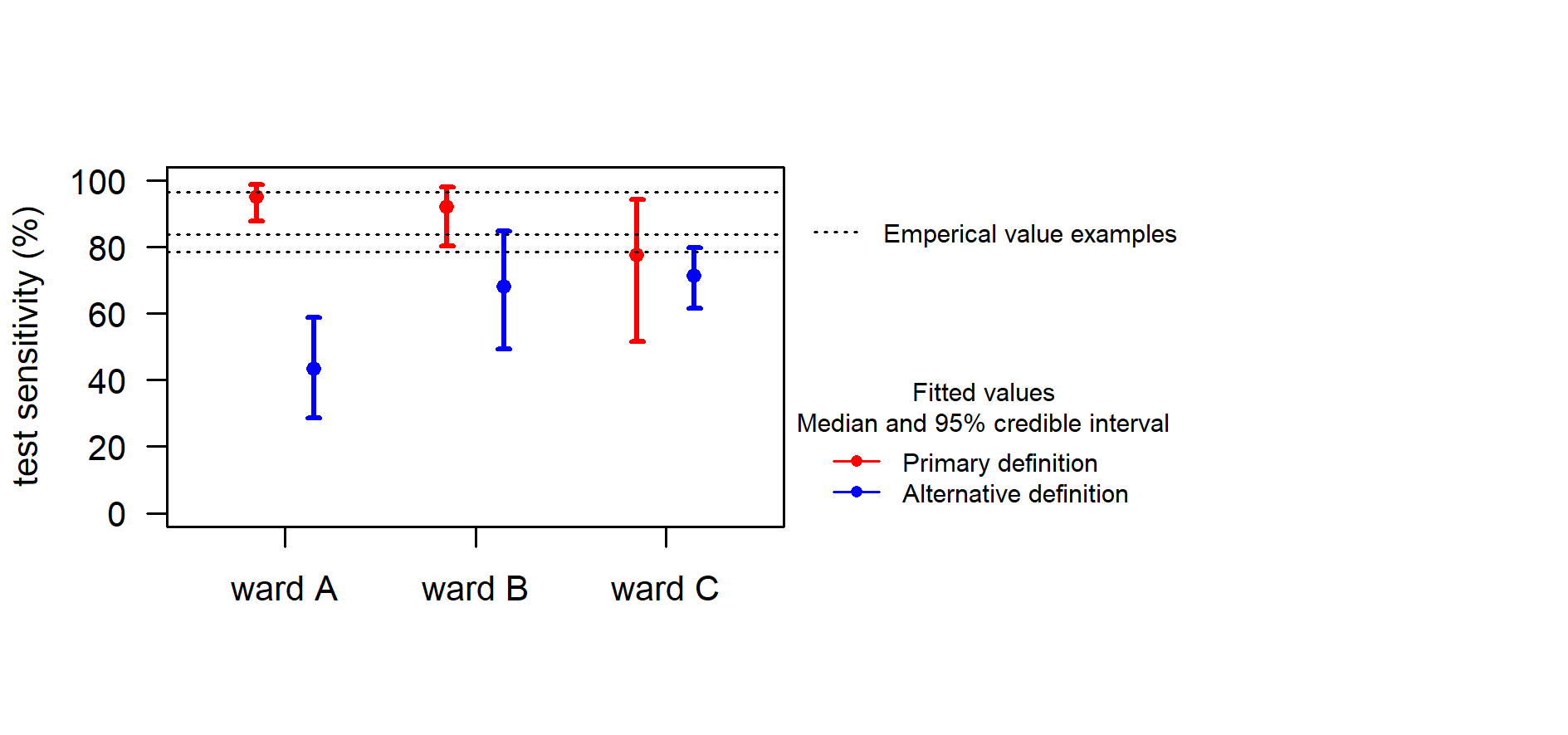 |

***a) marginal distributions of fitted beta coefficients. b) fitted test sensitivity values compared with example empirical values for a range of test methodologies***

### Clearance estimation

The rate of clearance of CPE - which was assumed to only occur within the community - was estimated from longitudinal data linking hospital admissions and test data from an England acute hospital trust^6^. Contiguous episodes from the admission data were combined to form hospital spells. Data were extracted for patients with pairs of consecutive spells separated by at least one night, defining a period in the community between those spells.

Data were retained for analysis where linked test data enabled an inference of colonisation status both at discharge and at re-admission (**Additional Figure 5a**) either side of the community period. CPE colonisation status at discharge was inferred from the last test sample date during the spell before the community period. Colonisation status at admission was inferred from samples taken within two days of re‑admission, with any CPE positive result inferring the patient was colonised (if no CPE-tested samples were taken in this period the community period was excluded). Samples taken later in the spell were not used for admission status inference, because after this time positive test results are conventionally attributed to hospital acquisition during the current spell^37, 38^.

Additional Figure 5. Clearance analysis

| ***a)***  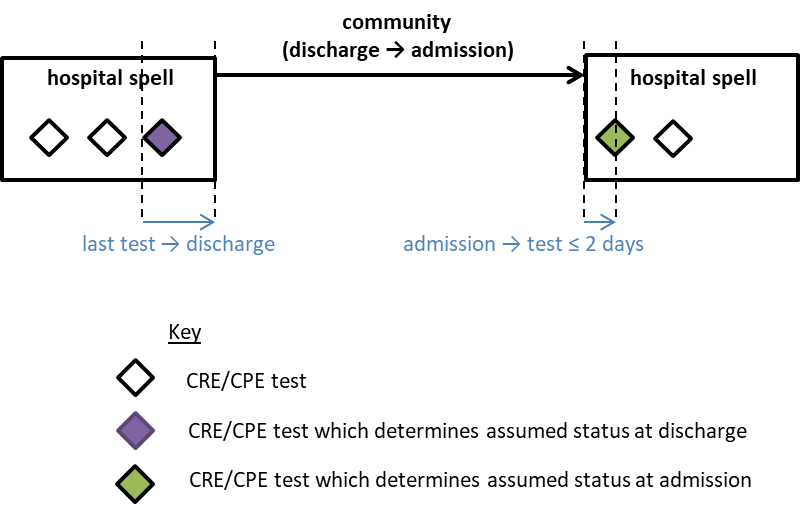 | ***b)***  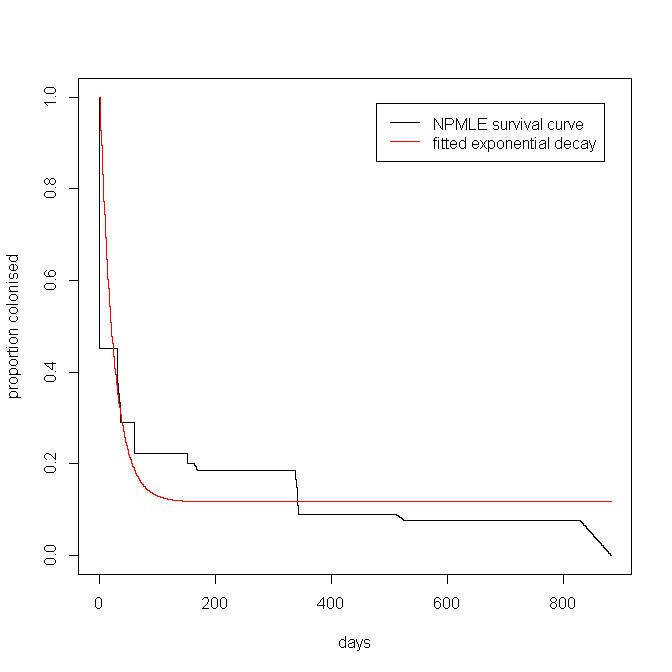 |
| --- | --- |

***a) inference of colonisation status at discharge and at admission. b) probability of colonisation at admission vs time in community – for readmitted patients discharged who were colonised at discharge***

A survival analysis was conducted on the subset of community spells where the patient was inferred as positive on entering the community (i.e. status at discharge). It was assumed that clearance could occur in the community, but not re-colonisation. Hence the status on readmission provided current status data on the time until clearance in the community (as measured by negative CPE test). Non‑parametric maximum likelihood estimation (NPMLE) was used to estimate the survival curve of CPE colonisation. The analysis was conducted in R (R version 3.5.1^49^ ) using icenReg package (version 2.0.9)^50^ which implements Turnbull’s Estimator^51^.

For incorporation in the model, a continuous distribution was generated by fitting exponential decay to the stepped survival curve using least squares. To avoid an assumption that colonisation cannot be permanent, a function representing exponential decay to an asymptotic value was used i.e.

$$p\left( n \right)=\alpha+\left( 1-\alpha\right)e^{-\lambda n}$$

and gives the probability that a patient, who was discharged from hospital in a colonised state, is still colonised on admission 𝑛 days later (𝑛 ∊ ℕ^+^). The estimated survival function and the fitted function are shown in **Additional Figure 5b**.

The survival analysis and function fitting were also conducted for colonisation status inference using the ‘CP-based’ test definition. However, the survival curve obtained indicated a median time to clearance of one day, which was not consistent with expert opinion on clinical experience of untreated CPE colonisation. Attempts to fit standard parametric distributions (exponential, Weibull, gamma, lognormal) obtained similarly implausible results with this (subsequently rejected) test definition.

## CPE infection prevention and control

### Screening, contact tracing and clinical tests sensitivity and specificity

Test methodology for the screening and confirmation tests assumed the modal type and method in use in England for CPE testing, with corresponding test sensitivity and specificity parameters taken from published literature. The type of test used for initial local and confirmation testing was informed by a 2016 survey of 50 laboratories and 26 NHS trusts in England^39^, cross-referenced with commentary in UK Standards for Microbiology Investigations^52^, and parameterisation was based on the modal method in use for each test type (identified from a survey of 121 NHS England laboratories in 2018^3^ of tests used for the detection and identification of carbapenemase^[[1]](#footnote-2)^‡).

Hence the swab test was assumed to be agar culture, parameterised using data for MacConkey selective agar plus carbapenem disc(s) as applied to rectal swab samples. The survey indicated that both phenotypic and molecular tests were in use for confirmatory testing; however for parameterisation purposes these tests were able to be assumed equivalent as sensitivity and specificity for the identified molecular method may be used as proxy for the identified phenotypic test^53^. The clinical test was assumed to be a locally-conducted automated susceptibility test, parameterised using the modal method^3^ for susceptibility testing including use for blood culture samples).

Each test applied was assumed to be independent from other tests, with outcome conditional only on colonisation status, i.e. the sensitivity and specificity for test application are not influenced by the outcomes of any other tests.

# Additional parameters

The transmission parameter estimates used for the patient-only transmission assumptions scenario are listed in **Additional Table 3**.

Additional Table 3. Transmission parameters assuming only patient transmission sources

| Transmission parameter | | From unknown sources  (𝛽_0_) | Per infectious patient on ward  (𝛽_1_) |
| --- | --- | --- | --- |
| Ward A | minimum  median  maximum | 0  0  0 | 0.00020254  0.00045806  0.00079047 |
| Ward B | minimum  median  maximum | 0  0  0 | 0.00001193  0.00027034  0.00077056 |
| Ward C | minimum  median  maximum | 0  0  0 | 0.00002684  0.00043957  0.00159457 |

# Model validation

Two steps in model validation were performed: code and output validation.

Model coding was validated, where possible, by using toy scenarios. These scenarios were chosen to isolate different model dynamics through choice of toy parameter values (e.g. 0 or 1 for probabilities and proportions) with effectively deterministic output for a specified variable. Analytical values were compared with model output under the corresponding toy scenario.

For output validation, individual trusts were modelled and simulation output for number of CPE positive records compared with values from a national surveillance system containing trust level data. The trusts used were a sample of convenience from those participating in the CPE ERS surveillance system^35^, excluding those with below average patient numbers (to minimise random effects). In these simulations, values from the referral-region hospital parameterisation were inherited, except for trust-specific estimates (using the same methodology as above) of hospital size, admission geography profiles, length of stay distributions (for spells ending with patient discharge and patient death). Figures reported in the ERS surveillance data for CPE positives were compared with the range of values in the model output for annual mean of CPE positive confirmations (from the range of transmission parameters). Validation was restricted to order of magnitude, given very limited data points were available from the surveillance data.

# Simulation results for region-typical hospitals

The simulations for region-typical hospitals used the following region-specific patient population and movement parameters: proportions by origin of admission, distributions of length of stay (until discharge and until death), prevalence on admission for local origin patients, distribution of maximum number of readmissions for local origin patients, distribution of time between admissions for readmissions, prevalence on admission for low-risk area and high-risk area origin patients. Parameters were estimated using the same methods as for the generic hospitals, but with the corresponding region acting as the local source geography, and the screening criteria strategy risk assumption for local patients for each region is shown in **Additional Table 1**. **Additional Figure 6** shows, for each of the 14 region-typical hospitals, the accuracy of categorisation by the Toolkit screening selection criteria for colonised and uncolonised admissions.

Additional Figure 6: Accuracy of Toolkit screening criteria categorisation for colonised and uncolonized admissions in region-typical hospitals

| 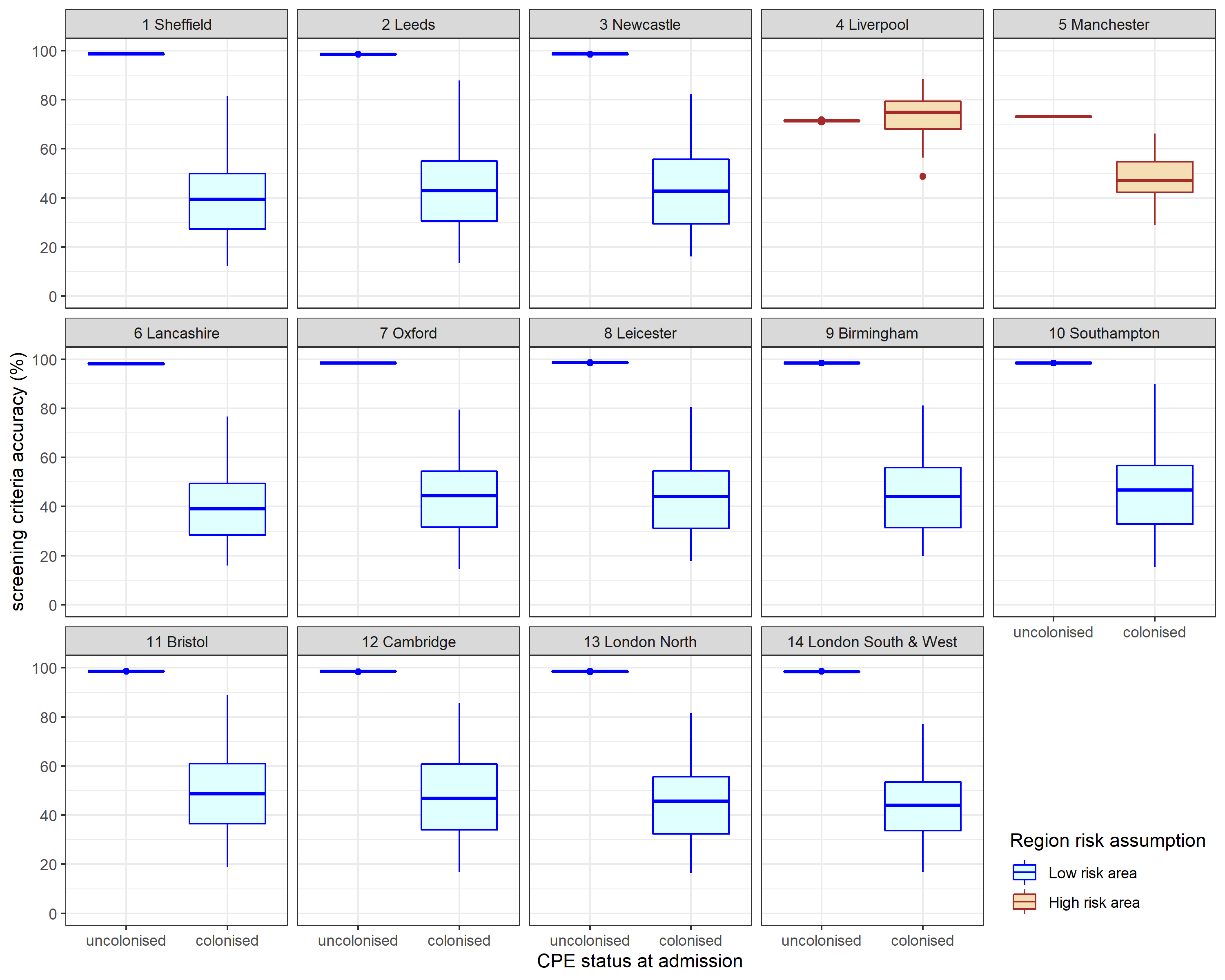 |
| --- |

**REFERENCES**

See main manuscript

1. ‡ Further survey detailed results included in personal communication from Freeman (Public Health England) 2018 [↑](#footnote-ref-2)
